# Corilagin alleviates atherosclerosis by inhibiting NLRP3 inflammasome activation via the Olfr2 signaling pathway *in vitro* and *in vivo*

**DOI:** 10.3389/fimmu.2024.1364161

**Published:** 2024-05-13

**Authors:** Jinqian Mao, Yunfei Chen, Qiushuo Zong, Cuiling Liu, Jiao Xie, Yujie Wang, David Fisher, Nguyen Thi Thu Hien, Khrystyna Pronyuk, Erkin Musabaev, Yiqing Li, Lei Zhao, Yiping Dang

**Affiliations:** ^1^Department of Vascular Surgery, Union Hospital, Tongji Medical College, Huazhong University of Science and Technology, Wuhan, China; ^2^Department of Infectious Diseases, Union Hospital, Tongji Medical College, Huazhong University of Science and Technology, Wuhan, China; ^3^Department of Medical Biosciences, Faculty of Natural Sciences, University of The Western Cape, Cape Town, South Africa; ^4^Hai Phong University of Medicine and Pharmacy, Haiphong, Vietnam; ^5^Department of Infectious Diseases, O.Bogomolets National Medical University, Kyiv, Ukraine; ^6^Research Institute of Virology, Ministry of Health, Tashkent, Uzbekistan

**Keywords:** atherosclerosis, inflammation, Olfr2, NLRP3 inflammasome, corilagin, therapeutic strategies

## Abstract

**Introduction:**

Atherosclerosis, a leading cause of global cardiovascular mortality, is characterized by chronic inflammation. Central to this process is the NOD-like receptor pyrin domain containing 3 (NLRP3) inflammasome, which significantly influences atherosclerotic progression. Recent research has identified that the olfactory receptor 2 (Olfr2) in vascular macrophages is instrumental in driving atherosclerosis through NLRP3- dependent IL-1 production.

**Methods:**

To investigate the effects of Corilagin, noted for its anti-inflammatory attributes, on atherosclerotic development and the Olfr2 signaling pathway, our study employed an atherosclerosis model in ApoE^−/−^ mice, fed a high-fat, high-cholesterol diet, alongside cellular models in Ana-1 cells and mouse bone marrow-derived macrophages, stimulated with lipopolysaccharides and oxidized low-density lipoprotein.

**Results:**

The vivo and vitro experiments indicated that Corilagin could effectively reduce serum lipid levels, alleviate aortic pathological changes, and decrease intimal lipid deposition. Additionally, as results showed, Corilagin was able to cut down expressions of molecules associated with the Olfr2 signaling pathway.

**Discussion:**

Our findings indicated that Corilagin effectively inhibited NLRP3 inflammasome activation, consequently diminishing inflammation, macrophage polarization, and pyroptosis in the mouse aorta and cellular models via the Olfr2 pathway. This suggests a novel therapeutic mechanism of Corilagin in the treatment of atherosclerosis.

## Introduction

Cardiovascular diseases, the leading cause of death globally, represent a severe health burden across both the high-income and developing nations (1–3). Atherosclerosis is the dominant risk factor for cardiovascular events including myocardial infarction, strokes, and peripheral arterial disease (4). Although various treatments exist for arteriosclerosis, including lipid-lowering and anti-platelet aggregation therapy, issues such as drug resistance, intolerance, and increased risk of sever bleeding events are prevalent (3, 5–7). Atherosclerosis is recognized as a chronic inflammatory condition characterized by the presence of immune cells in lesions, which produce a range of pro-inflammatory cytokines (8, 9). Macrophages, the pro-inflammatory cells, make significant contributions to the development of atherosclerosis (10). Normal and modified lipoproteins are accumulated in macrophages, promoting foam cell formation and activation of innate immune receptors likes NOD-like and Toll-like receptors (NLRs and TLRs) to exacerbate inflammation and plaque progression (11, 12).

The NOD-like receptor pyrin domain containing 3 (NLRP3) inflammasome, a complex of multimeric cytosolic proteins, is pivotal in atherosclerotic progression (2, 8, 13, 14). Additionally, dysregulation of TLRs is a crucial mechanism for inflammation and atherosclerosis, primarily due to their role in regulating the expression of key molecules (8, 15–17). The activation of the NLRP3 inflammasome in macrophages typically requires two distinct signals: priming and activation (2, 8, 18). During priming, TLRs or cytokine receptors, stimulated by damage-associated and pathogen-associated molecular patterns, transcriptionally upregulate NLRP3, pro-IL-18, and pro–IL-1β via NF-κB signaling, thus facilitates NLRP3-mediated inflammasome assembly (2, 8, 19–21). In the activation phase, factors such as phagolysosomal rupture, disrupted ion homeostasis, mitochondrial damage, or reactive oxygen species are identified as common triggers for NLRP3 activation (2, 22–24). Subsequently, NIMA-related kinase 7 (NEK7) interacts with NLRP3 to recruit pro-caspase-1 through the apoptosis-associated speck-like protein containing a caspase recruitment domain (ASC). Activated caspase-1 then converts pro-IL-1β and pro-IL-18 into their active forms, also triggering gasdermin D (GSDMD) production (2, 25–28). NLRP3 inflammasome activation could cause the overexpression of inflammatory factors involved in atherosclerosis progression (8, 29), the polarization of macrophages into M1 type that fosters tissue destruction and secretion of pro-inflammatory factors (30, 31), and the increasing expression of GSDMD resulting in pyroptosis to enhance vascular permeability and membrane damage (32–34). Recently, Orecchioni et al. have revealed that olfactory receptor 2 (Olfr2) interacts with TLR4 in vascular macrophages leading to NLRP3 inflammasomes activation via adenylate cyclase 3 (Adcy3) to drive atherosclerosis development, provides a new target for the prevention and treatment of atherosclerosis (35).

Corilagin, a polyphenolic monomer isolated from Phyllanthus urinaria, exhibits diverse pharmacological properties including antitumor, antioxidant, and anti-inflammatory effects (17, 36). It can ameliorate inflammatory lesions in macrophages by inhibiting NLRP3 inflammasome activation and pyroptosis (37, 38). Nonetheless, the specific impact of Corilagin on NLRP3 inflammasome activation in atherosclerosis necessitates further investigation. TLR4 can provide the priming signal of NLRP3 inflammasome activation via NF-κB pathway (12). Several researches have indicated Corilagin’s potential in preventing and treating atherosclerosis (17, 39–42), and find Corilagin probably mitigate atherosclerosis by inhibiting the TLR4 signaling pathway (17, 40). However, its regulatory mechanisms remain unclear, necessitating further investigation into its anti-atherosclerotic efficacy. Consequently, we hypothesize that Corilagin can suppress the activation of NLRP3 inflammasome to alleviate atherosclerosis by disrupting the Olfr2 signaling pathway. This study aims to examine the potential effects and mechanisms of Corilagin on atherosclerosis through the Olfr2 pathway, employing treatments on Ana-1 cells, primary mouse bone marrow-derived macrophages (BMDMs), and atherosclerotic models in ApoE^−/−^ mice.

## Materials and methods

### Reagents

Corilagin standard substance (SC9500, purity ≥ 98%) for cells and aspirin for animals (A8830, purity ≥ 98%) were purchased from Solarbio (Beijing, China). Corilagin for animal experimentation (N0272, purity 90% ~ 99%) was obtained from Hengcheng Zhiyuan Biotechnology (Sichuan, China). Aspirin for cells (B21505, purity ≥ 98%) was purchased from Yuanye Biotechnology (Shanghai, China). Oxidized low-density lipoprotein (Ox-LDL) was purchased from Yiyuan Biotechnology (YB-002, Guangzhou, China). Lipopolysaccharides (LPS, L8880) was purchased from Solarbio. Fetal bovine serum (FBS, 10099–141) and Roswell Park Memorial Institute (RPMI)-1640 were obtained from Gibco (New York, USA). Phosphate buffer saline (PBS) was obtained from Servicebio (G4202, Wuhan, China) and 4% paraformaldehyde (PFA) was purchased from Biosharp (BL520, Guangzhou, China).

### Cell culture and infection

The Ana-1 cell line, a murine macrophage line Ana-1 cells, was obtained from the Type Culture Collection of the Chinese Academy of Sciences (Shanghai, China). Cells were cultured in RPMI-1640 medium containing 10% FBS and maintained at 37°C in a saturated humidity incubator with 5% CO2. The lentivirus of short hairpin (Sh)-Olfr2 (Sh-Olfr2), Olfr2-overexpression (Olfr2-OE), Sh-Control (Sh-Ctrl), and Control-OE (Ctrl-OE) were constructed by GeneChem (Shanghai, China). Ana-1 cells were planted into 6‐well plates with a density of 1×10^5^/well for 24h and then infected with lentivirus with a multiplicity of infection of 40 according to our previous experiment (17). The medium was replaced within 12h after infection and were cultured for an additional 72h. Then, 2µg/ml of puromycin (Sigma–Aldrich, St. Louis, MO, USA) was added to the complete medium to kill the wild-type cells for 2 weeks, and the culture medium was changed every 2 days. The efficiency of infection was determined by quantitative real-time PCR (RT-qPCR). We cultured the remaining lentivirus‐infected cells for further experimentations.

### BMDMs generation

The femora and tibiae from C57BL/6J were flushed with 5ml ice cold RPMI-1640 medium to isolate mouse bone marrow. Bone marrow cells were filtered by a 70µm cell strainer (BS-70-XBS, Biosharp) and red blood cell lysis buffer (R1010, Solarbio) was utilized to lyse red blood cells. The remaining cells were cultured in RPMI-1640 medium supplemented with 20ng/ml of mouse recombinant macrophage-colony stimulating factor (315–02, Peprotech, NJ, USA) for 7 days. The culture medium was changed every 2 days to obtain BMDMs for further studies.

### Cell stimulation and treatment

Ana-1 Cells or BMDMs were seeded at 6-well plates overnight. They were divided into 6 groups: the Control group, the Model group, the Aspirin group (Positive control group), the Corilagin (100μg/ml) group, the Corilagin (50μg/ml) group, and the Corilagin (25μg/ml) group. Except the Control group, the other groups were stimulated with LPS (1μg/ml) and Ox-LDL (100μg/ml) for 24h. Subsequently, the Aspirin group and the Corilagin (100, 50 and 25μg/ml) groups were treated with aspirin and different concentrations of Corilagin. In addition, the lentivirus‐infected cells were grown on 6-well plates for 24h and divided into 8 groups: the Sh-Ctrl group, the Sh-Olfr2 group, the Sh-Ctrl+Corilagin (100μg/ml) group, the Sh-Olfr2+Corilagin (100μg/ml) group, the Ctrl-OE group, the Olfr2-OE group, the Ctrl-OE+Corilagin (100μg/ml) group, and the Olfr2-OE+Corilagin (100μg/ml) group. After all groups stimulating by LPS+Ox-LDL for 24h, the Sh-Ctrl+Corilagin group, the Sh-Olfr2+Corilagin group, the Ctrl-OE+Corilagin group, and the Olfr2-OE+Corilagin group were treated with Corilagin. After treating with Corilagin for 24h in our study, cell culture medium and cell lysates were gathered for subsequent experiments (**Supplementary Figure 1**).

### Animal treatments and sample collection

All animal experimental protocols are approved by the Animal Care and Use Committee of Bainte Biotechnology (Wuhan, China) [[2023] IACUC number: 004]. ApoE^−/−^ mice (male, 5 weeks, 18–24g) were provided by Bainte Biotechnology and exposed to a 12-h light/dark cycle in plenty of water and food. After a week of adaptive feeding, mice were randomly divided into 14 groups with six mice in each group: the Control group, the Model group, the Aspirin group, the Corilagin (40mg/kg) group, the Corilagin (20mg/kg) group, the Corilagin (10mg/kg) group, the Sh-Ctrl group, the Sh-Olfr2 group, the Sh-Ctrl+Corilagin group, the Sh-Olfr2+Corilagin group, the Ctrl-OE group, the Olfr2-OE group, the Ctrl-OE+Corilagin group, and the Olfr2-OE+Corilagin group. The Control group was fed a normal diet while other groups were fed a high fat and cholesterol diet (D12108C; HFK Bios) for 8 weeks. After feeding 8 weeks, the Sh-Ctrl group, the Sh-Olfr2 group, the Sh-Ctrl+Corilagin (40mg/kg) group, the Sh-Olfr2+Corilagin (40mg/kg) group, the Ctrl-OE group, the Olfr2-OE group, the Ctrl-OE+Corilagin (40mg/kg) group, and the Olfr2-OE+Corilagin (40mg/kg) group were injected lentivirus (4×10^7^Tfu, 20μl) via tail vein. Simultaneously, the same amount of physiologic (0.9%) saline were gave in the Control group, the Model group, the Sh-Ctrl group, the Sh-Olfr2 group, the Ctrl-OE group and the Olfr2-OE group, and other groups were treated with aspirin or different concentrations of Corilagin through intragastric administration every 2 days for 2 weeks (**Supplementary Figure 2**). Finally, mice were sacrificed and perfused with PBS. Aortas were divided into two segments: the vessel closed to aortic root was reserved in 4% PFA for hematoxylin and eosin (HE), Oil Red O, and immunohistochemical (IHC) staining, whereas the other section was placed at -80°C for RT-qPCR and western blotting (WB) analysis. Blood samples were collected by removing mouse eyeballs. Serum was separated by centrifugation at 4°C (2500rpm, 15min) and stored at -80°C for serum lipids detection and Enzyme-Linked Immunosorbent Assay (ELISA) analysis.

### Quantitative real-time PCR analysis

The mRNA expressions of Olfr2, iNOS, Adcy3, NLRP3, GSDMD, Caspase-1, Arg-1, NEK7, ASC, IL-1β, IL-18, and TNF-α were measured by RT-qPCR. Total RNA was isolated from Ana-1 cells, BMDMs, or aortas by Trizol reagent (R401–01, Vazyme, Nanjing, China). The isolated total RNA was reverse transcribed into complimentary (c)DNA via the HiScript II Q RT SuperMix (R223–01, Vazyme). The RT-qPCR was performed on StepOne™ Plus device (Applied Biosystems, Foster City, CA, USA) following the protocol of HiScript II One Step RT-qPCR SYBR Green Kit (Q221–01, Vazyme). The 2^-ΔΔCT^ method was used to analyze the relative mRNA expression normalized to the reference gene GAPDH. All primers were synthesized by TSINGKE (Wuhan, China). The primer sequences of genes were presented in **Table 1**.

**Table 1:** Primer sequences used in this study.

Primer	Forward	Reverse
Olfr2	CTTGCTGGCTTCATTGGTTCCG	CCATGACAGCAAGAAGGACACAC
Adcy3	CCAACTTTGCTGACTTCTACAC	TGTCCAGGAGAGAGTCAAAATC
NEK7	GCTGTCTGCTATATGAGATGGC	CCGAATAGTGATCTGACGGGAG
NLRP3	ATTACCCGCCCGAGAAAGG	TCGCAGCAAAGATCCACACAG
ASC	GCTACTATCTGGAGTCGTATGGC	GACCCTGGCAATGAGTGCTT
Caspase-1	CACAGCTCTGGAGATGGTGA	GGTCCCACATATTCCCTCCT
GSDMD	CCATCGGCCTTTGAGAAAGTG	ACACATGAATAACGGGGTTTCC
IL-1β	TTCAGGCAGGCAGTATCACTC	GAAGGTCCACGGGAAAGACAC
IL-18	GACTCTTGCGTCAACTTCAAGG	CAGGCTGTCTTTTGTCAACGA
Arg-1	CTCCAAGCCAAAGTCCTTAGAG	AGGAGCTGTCATTAGGGACATC
iNOS	CCCTTCAATGGTTGGTACATGG	ACATTGATCTCCGTGACAGCC
TNF-α	CAGGCGGTGCCTATGTCTC	CGATCACCCCGAAGTTCAGTAG
GAPDH	GAGCAAGGACACTGAGCAAGA	GCCCCTCCTGTTATTATGGGG

### Western blotting analysis

The protein expressions of iNOS (A3774, Abclonal), Adcy3 (19492–1-AP, Proteintech), NLRP3 (ab270449, Abcam), GSDMD (66387–1-Ig, Proteintech), Caspase-1 (22915–1-AP, Proteintech), Arg-1 (16001–1-AP, Proteintech), NEK7 (A19816, Abclonal), and ASC (67824, Cell Signaling Technology) were measured by WB. Total protein was isolated from Ana-1 cells, BMDMs, or aortas by RIPA lysis buffer and protease inhibitor (P0013B and P1005, Beyotime, Shanghai, China), and quantified according to the instructions of the BCA Protein Assay Kit (P0012, Beyotime). The protein samples were separated by 10% sodium dodecyl sulfate–polyacrylamide gel electrophoresis and transferred to polyvinylidene difluoride membranes. Membranes were blocked with 5% non-fat milk in Tris-buffered saline and Tween 20 (TBST) for 1h and incubated with primary antibodies at 4°C overnight. After washing thrice with TBST, membranes were incubated with horseradish peroxidase-conjugated secondary antibody (SA00001–1 and SA00001–2, Proteintech, Wuhan, China) for 1h at room temperature (RT). These membranes were exposed in a dark room via electrochemiluminescence reagent (BL520, Biosharp). Finally, the grayscale values were measured by ImageJ software to quantify the WB bands contracted with GAPDH (60004–1-Ig, Proteintech).

### Serum lipids and inflammatory cytokines analysis

Triglyceride (TG), Total cholesterol (TC), high-density lipoprotein cholesterol (HDL-C), and low-density lipoprotein cholesterol (LDL-C) in the serum of mice were detected by an automatic biochemical analyzer (Shenzhen Leidu Life Sciences, China). The level of IL-1β, IL-18, and TNF-α in the mouse serum or cell supernatant was measured through ELISA Kits from Ruixin Biotechnology (Quanzhou, China).

### Histological analysis

Sections were stained with HE to visualize pathological changes in the vascular tissue and Oil red O staining was applied for lipid plaques analysis. Images were captured by an electron microscope (Olympus, Tokyo, Japan) and analyzed by ImageJ software. Besides, Olfr2 expression in animal model was examined through IHC staining. The tissue sections were stained with Olfr2 antibody (1:500, OSR00025G, Thermo Fischer) followed by Goat Anti-Rabbit IgG (1:200, G1213, Servicebio, Wuhan, China). The sections were incubated with 3,3-diaminobenzidine and counterstained with hematoxylin. Finally, images were captured by an automatic scanning microscope (Zeiss, Germany) and analyzed via ImageJ software.

### Flow cytometric analysis

Cluster of differentiation (CD) 86 and CD206 expressions were measured by flow cytometric (FC) analysis. Ana-1 cells or BMDMs treated as indicated above were incubated with Mouse Fc block™ solution (553141, BD Biosciences, San Jose, CA, USA) for 5min at 4°C. Then they were stained with fluorescein isothiocyanate anti-F4/80 antibody (123107, BioLegend, San Diego, CA, USA), phycoerythrin anti-cluster of CD 86 antibody (E-AB-F099D), and peridinin chlorophyll protein/Cyanine5.5 anti-CD11b antibody (E-AB-F1081J, Elabscience, Wuhan, China) for 30 min at 4°C. All cells were washed two times with cold PBS and incubated with BD Cytofix/Cytoperm™ Fixation and Permeabilization Solution (554722) for 30min at 4°C. In order to permeabilization, the fixed cells were washed two times and incubated in 1X BD Perm/Wash™ buffer (554723) for 15min at 4°C. The fixed/permeabilized cells were thoroughly suspended in 100µL of 1X BD Perm/Wash™ buffer and stained with allophycocyanin anti-CD206 antibody (141707, BioLegend) in the dark for 30min at RT. After washing twice with PBS, cell suspensions were analyzed on a BD FACSCalibur™ flow cytometer and calculated by FlowJo software (Treestar Inc).

### Immunofluorescence analysis

The Olfr2 expression was performed by immunofluorescence (IF) analysis. Ana-1 cells or BMDMs were cultured on 14 mm round coverslips (BS-14-RC, Biosharp) in the 24-well plate (703001, Nest) and treated as described above. Cells were fixed with 4% PFA for 10min at RT. After washed three times with cold PBS, samples were incubated with 5% BSA (G1208, Servicebio) for 10min at RT and maintained in Olfr2 antibody (1:500, Thermo Fischer) at 4°C overnight. Subsequently, cells were washed three times and stained with secondary antibody (1:200, GB25303, Servicebio) for 1h at RT. DAPI (G1012, Servicebio) was used to stain nuclei in the dark for 10min. After washed three times, coverslips were sealed with anti-fluorescence quenching reagent (G1401, Servicebio) on glass slides. Images were captured by Zeiss scanning microscope and analyzed via ImageJ software.

### Statistical analysis

All statistical analyses were performed by using GraphPad Prism 9.0 software. Data were presented as the mean ± standard deviation (SD). Shapiro-Wilk test was performed to assess the normalcy, and *p* > 0.05 was defined as normally distributed. Brown-Forsythe test was performed to assess the equality of variances, and *p* > 0.05 was defined as equal variances. Student’s t-test was performed to compare data between two groups and one-way analysis of variance (ANOVA) test was performed for multiple comparisons, and *p* < 0.05 was defined as statistically significant.

## Result

### Effects of corilagin on atherosclerosis *in vivo*

**Figure 1:**
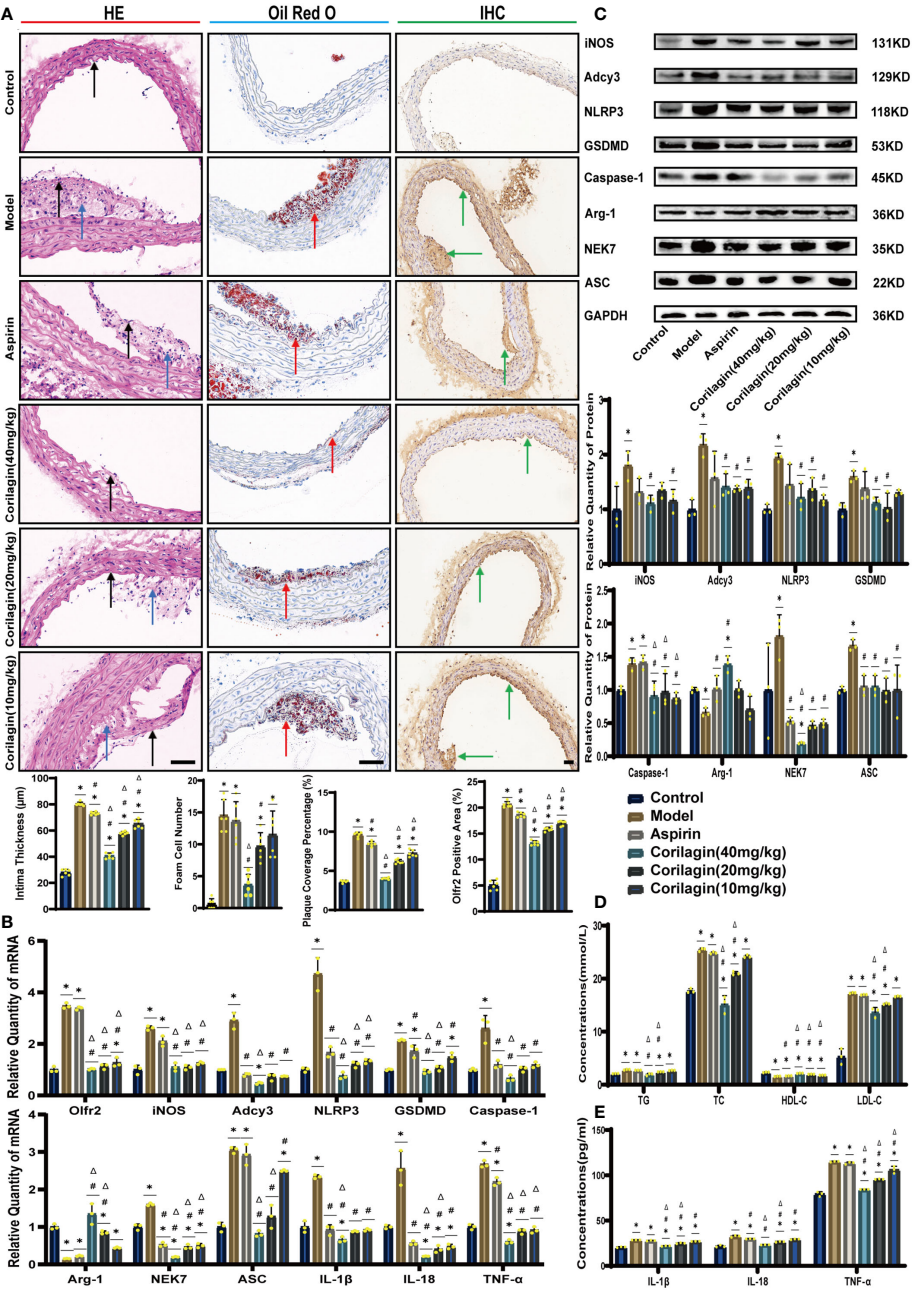
Effects of Corilagin on atherosclerosis and Olfr2 signal pathway *in vivo*. **(A)** Pathological changes, plaque coverage percentage, and Olfr2 expression in mouse aorta were visualized by HE, Oil red O, and IHC staining respectively and quantitative analyses of intima thickness and foam cell number based on HE, plaque coverage percentage based on Oil red O, and Olfr2 expression based on IHC. Black arrows indicated aorta intima and blue arrows indicated foam cell and fibrous tissue in HE. Yellow arrows indicated lipid plaque in Oil red O. Green arrows indicated the area of Olfr2 expression in IHC. ^*^p < 0.05 compared with the Control group, ^#^*p* < 0.05 compared with the Model group, ^Δ^*p* < 0.05 compared with the Aspirin group determined by one-way ANOVA test (n = 6). Data was presented as the mean ± SD. Scale bars, 50μm. **(B)** mRNA expression of Olfr2, Adcy3, NLRP3, Caspase-1, ASC, NEK7, GSDMD, IL-1β, IL-18, Arg-1, iNOS, and TNF-α in mouse aorta was measured by RT-qPCR. ^*^p < 0.05 compared with the Control group, ^#^*p* < 0.05 compared with the Model group, ^Δ^*p* < 0.05 compared with the Aspirin group determined by one-way ANOVA test (n = 3). Data was presented as the mean ± SD. **(C)** Protein expression of iNOS, Adcy3, NLRP3, GSDMD, Caspase-1, NEK7, Arg-1, and ASC in mouse aorta was measured by WB and quantitative analyses of protein level based on WB. ^*^p < 0.05 compared with the Control group, ^#^*p* < 0.05 compared with the Model group, ^Δ^*p* < 0.05 compared with the Aspirin group determined by one-way ANOVA test (n = 3). Data was presented as the mean ± SD. **(D)** Serum lipids of mice were detected by an automatic biochemical analyzer. ^*^p < 0.05 compared with the Control group, ^#^*p* < 0.05 compared with the Model group, ^Δ^*p* < 0.05 compared with the Aspirin group determined by one-way ANOVA test (n = 3). Data were presented as the mean ± SD. **(E)** IL-1β, IL-18, and TNF-α in the mouse serum were measured by ELISA. ^*^p < 0.05 compared with the Control group, ^#^*p* < 0.05 compared with the Model group, ^Δ^*p* < 0.05 compared with the Aspirin group determined by one-way ANOVA test (n = 3). Data was presented as the mean ± SD.

To investigate the impact of Corilagin on atherosclerotic lesion formation, we established an ApoE^−/−^ mouse model for *in vivo* experiments. HE staining, as depicted in **Figure 1A**, demonstrated that the aorta intima in the Model group was notably deformed and thickened, with the formation of plaques predominantly composed of foam cells and fibrous tissue. In contrast with the Model or Aspirin group, the Corilagin (40mg/kg) group exhibited a reduction in intima thickness and foam cell numbers. In addition, the percentage of red lipid plaques in the Corilagin groups was lower than that in both the Model and Aspirin group (**Figure 1A**). Furthermore, serum lipid levels of TG, TC and LDL-C in the Corilagin (20 and 40mg/kg) groups were reduced, while HDL-C levels were elevated compared to both the Model and Aspirin group (**Figure 1D**). These results indicated that Corilagin effectively mitigates atherosclerotic lesions in this animal model.

### Effects of corilagin on Olfr2 and downstream molecules *in vivo* and vitro

**Figure 2:**
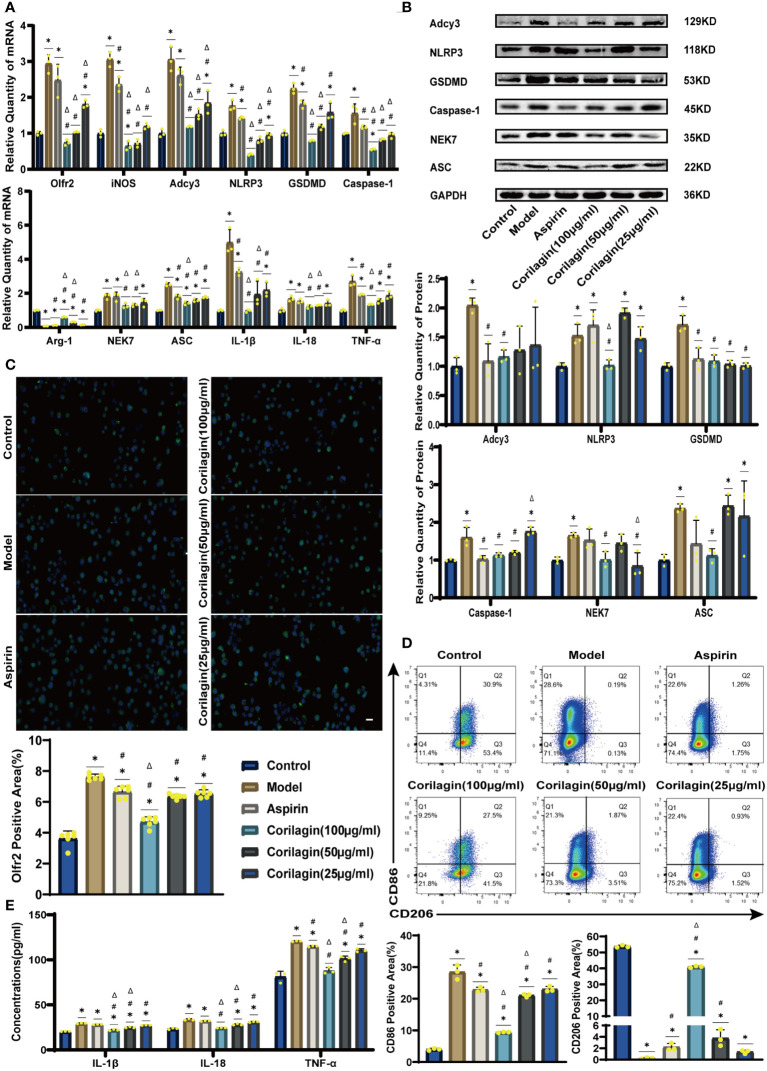
Effects of Corilagin on Olfr2 signal pathway in LPS+Ox-LDL-induced Ana-1 cells. **(A)** mRNA expression of Olfr2, Adcy3, NLRP3, Caspase-1, ASC, NEK7, GSDMD, IL-1β, IL-18, Arg-1, iNOS, and TNF-α in Ana-1 cells was measured by RT-qPCR. ^*^p < 0.05 compared with the Control group, ^#^*p* < 0.05 compared with the Model group, ^Δ^*p* < 0.05 compared with the Aspirin group determined by one-way ANOVA test (n = 3). Data was presented as the mean ± SD. **(B)** Protein expression of Adcy3, NLRP3, GSDMD, Caspase-1, NEK7, and ASC in Ana-1 cells was measured by WB and quantitative analyses of protein level based on WB. ^*^p < 0.05 compared with the Control group, ^#^*p* < 0.05 compared with the Model group, ^Δ^*p* < 0.05 compared with the Aspirin group determined by one-way ANOVA test (n = 3). Data was presented as the mean ± SD. **(C)** Olfr2 expression in Ana-1 cells was visualized by IF and quantitative analyses of Olfr2 level based on IF. ^*^p < 0.05 compared with the Control group, ^#^*p* < 0.05 compared with the Model group, ^Δ^*p* < 0.05 compared with the Aspirin group determined by one-way ANOVA test (n = 6). Data was presented as the mean ± SD. Scale bars, 20μm. **(D)** CD86 and CD206 expression in Ana-1 cells were measured by FC and quantitative analyses of CD86 and CD206 level based on FC. ^*^p < 0.05 compared with the Control group, ^#^*p* < 0.05 compared with the Model group, ^Δ^*p* < 0.05 compared with the Aspirin group determined by one-way ANOVA test (n = 3). Data was presented as the mean ± SD. **(E)** IL-1β, IL-18, and TNF-α in Ana-1 cell supernatant were measured by ELISA. ^*^p < 0.05 compared with the Control group, ^#^*p* < 0.05 compared with the Model group, ^Δ^*p* < 0.05 compared with the Aspirin group determined by one-way ANOVA test (n = 3). Data was presented as the mean ± SD.

**Figure 3:**
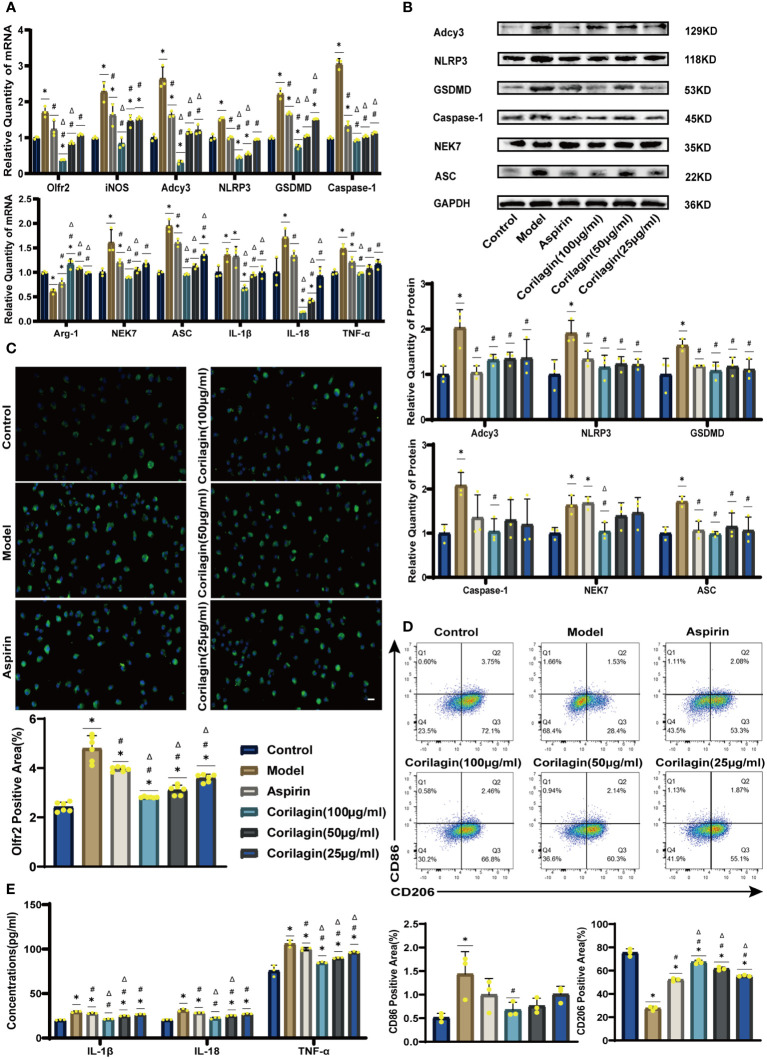
Effects of Corilagin on Olfr2 signal pathway in LPS+ox-LDL-induced primary mouse BMDMs. **(A)** mRNA expression of Olfr2, Adcy3, NLRP3, Caspase-1, ASC, NEK7, GSDMD, IL-1β, IL-18, Arg-1, iNOS, and TNF-α in primary mouse BMDMs was measured by RT-qPCR. ^*^p < 0.05 compared with the Control group, ^#^*p* < 0.05 compared with the Model group, ^Δ^*p* < 0.05 compared with the Aspirin group determined by one-way ANOVA test (n = 3). Data was presented as the mean ± SD. **(B)** Protein expression of Adcy3, NLRP3, GSDMD, Caspase-1, NEK7, and ASC in primary mouse BMDMs was measured by WB and quantitative analyses of protein level based on WB. ^*^p < 0.05 compared with the Control group, ^#^*p* < 0.05 compared with the Model group, ^Δ^*p* < 0.05 compared with the Aspirin group determined by one-way ANOVA test (n = 3). Data was presented as the mean ± SD. **(C)** Olfr2 expression in primary mouse BMDMs was visualized by IF and quantitative analyses of Olfr2 level based on IF. ^*^p < 0.05 compared with the Control group, ^#^*p* < 0.05 compared with the Model group, ^Δ^*p* < 0.05 compared with the Aspirin group determined by one-way ANOVA test (n = 6). Data was presented as the mean ± SD. Scale bars, 20μm. **(D)** CD86 and CD206 expression in primary mouse BMDMs were measured by FC and quantitative analyses of CD86 and CD206 level based on FC. ^*^p < 0.05 compared with the Control group, ^#^*p* < 0.05 compared with the Model group, ^Δ^*p* < 0.05 compared with the Aspirin group determined by one-way ANOVA test (n = 3). Data was presented as the mean ± SD. **(E)** IL-1β, IL-18, and TNF-α in primary mouse BMDMs cell supernatant were measured by ELISA. ^*^p < 0.05 compared with the Control group, ^#^*p* < 0.05 compared with the Model group, ^Δ^*p* < 0.05 compared with the Aspirin group determined by one-way ANOVA test (n = 3). Data was presented as the mean ± SD.

To explore the effect of Corilagin on Olfr2 expression, qRT-PCR, IHC and IF were performed to assess Olfr2 levels in aortic tissues and cellular models. Olfr2 expression was significantly reduced in the Corilagin (40mg/kg or 100µg/ml) group compared to both the Model and Aspirin group (**Figures 1A, 1B**, **2A, 2C**, **3A, 3C**). The reduction in Olfr2 expression in atherosclerotic mice and cellular models probably suggested a potential pathway through that Corilagin regulated atherosclerosis development. To elucidate the underlying molecular mechanisms of Corilagin’s anti-atherosclerotic effects, we further analyzed downstream molecules associated with Olfr2 *in vivo* and vitro experiments. The mRNA and protein levels of Adcy3, NLRP3 inflammasome effectors (NLRP3, Caspase-1, NEK7 and ASC) and the cell pyroptosis-related molecule (GSDMD) were lower in the Corilagin (40mg/kg or 100µg/ml) group than in the Model group (**Figures 1B, 1C**, **2A, 2B**, **3A, 3B**). In the Corilagin (40mg/kg) group, both mRNA and protein levels of iNOS in mouse aortic tissues were significantly reduced compared to the Model group, as evidenced in **Figures 1B, 1C**. Conversely, Arg-1 levels demonstrated a reverse pattern, indicating an anti-inflammatory effect. Similarly, in cellular models, the Corilagin treatment (100µg/ml) led to a reduction in M1 macrophage polarization markers (iNOS and CD86), while markers associated with M2 macrophage polarization (Arg-1 and CD206) were elevated, compared to the Model group (**Figures 2A, 2D**, **3A, 3D**). Additionally, inflammatory factors such as IL-1β, IL-18, and TNF-α measured via RT-qPCR and ELISA, showed reduced expression following Corilagin (40mg/kg or 100µg/ml) treatment when compared to the Model or Aspirin group (**Figures 1B, 1E**, **2A, 2E**, **3A, 3E**). Consequently, these results led us to hypothesize that Corilagin could suppress NLRP3 inflammasome activation and subsequent cytokine production, macrophage polarization, and cell pyroptosis by inhibiting the Olfr2 signaling pathway.

### Effects of corilagin on atherosclerosis after downregulating Olfr2 *in vivo*

**Figure 4:**
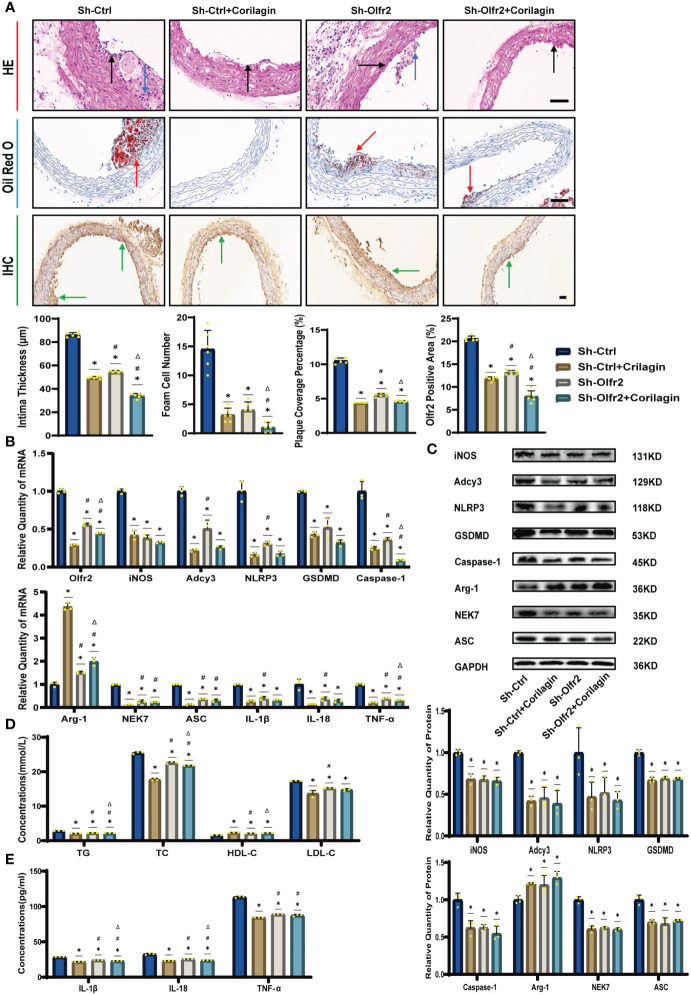
Effects of Corilagin on atherosclerosis and Olfr2 signal pathway after downregulating Olfr2 *in vivo*. **(A)** Pathological changes, plaque coverage percentage, and Olfr2 expression in mouse aorta were visualized by HE, Oil red O, and IHC staining respectively and quantitative analyses of intima thickness and foam cell number based on HE, plaque coverage percentage based on Oil red O, and Olfr2 expression based on IHC. Black arrows indicated aorta intima and blue arrows indicated foam cell and fibrous tissue in HE. Yellow arrows indicated lipid plaque in Oil red O. Green arrows indicated the area of Olfr2 expression in IHC. ^*^p < 0.05 compared with the Sh-Ctrl group determined by one-way ANOVA test (n = 6), ^#^*p* < 0.05 compared with the Sh-Ctrl+Corilagin group determined by one-way ANOVA test (n = 6), ^Δ^*p* < 0.05 compared with the Sh-Olfr2 group determined by Student’s t-test (n = 6). Data was presented as the mean ± SD. Scale bars, 50μm. **(B)** mRNA expression of Olfr2, Adcy3, NLRP3, Caspase-1, ASC, NEK7, GSDMD, IL-1β, IL-18, Arg-1, iNOS, and TNF-α in mouse aorta was measured by RT-qPCR. ^*^p < 0.05 compared with the Sh-Ctrl group determined by one-way ANOVA test (n = 3), ^#^*p* < 0.05 compared with the Sh-Ctrl+Corilagin group determined by one-way ANOVA test (n = 3), ^Δ^*p* < 0.05 compared with the Sh-Olfr2 group determined by Student’s t-test (n = 3). Data was presented as the mean ± SD. **(C)** Protein expression of iNOS, Adcy3, NLRP3, GSDMD, Caspase-1, NEK7, Arg-1, and ASC in mouse aorta was measured by WB and quantitative analyses of protein level based on WB. ^*^p < 0.05 compared with the Sh-Ctrl group determined by one-way ANOVA test (n = 3), ^#^*p* < 0.05 compared with the Sh-Ctrl+Corilagin group determined by one-way ANOVA test (n = 3), ^Δ^*p* < 0.05 compared with the Sh-Olfr2 group determined by Student’s t-test (n = 3). Data was presented as the mean ± SD. **(D)** Serum lipids of mice were detected by an automatic biochemical analyzer. ^*^p < 0.05 compared with the Sh-Ctrl group determined by one-way ANOVA test (n = 3), ^#^*p* < 0.05 compared with the Sh-Ctrl+Corilagin group determined by one-way ANOVA test (n = 3), ^Δ^*p* < 0.05 compared with the Sh-Olfr2 group determined by Student’s t-test (n = 3). Data was presented as the mean ± SD. **(E)** IL-1β, IL-18, and TNF-α in the mouse serum were measured by ELISA. ^*^p < 0.05 compared with the Sh-Ctrl group determined by one-way ANOVA test (n = 3), ^#^*p* < 0.05 compared with the Sh-Ctrl+Corilagin group determined by one-way ANOVA test (n = 3), ^Δ^*p* < 0.05 compared with the Sh-Olfr2 group determined by Student’s t-test (n = 3). Data was presented as the mean ± SD.

We specifically downregulated Olfr2 expression in the animal model to further demonstrate the mechanism behind Corilagin therapeutic effects. As depicted in **Figures 4A, 4D**, the Sh-Olfr2 group exhibited improvements in pathological changes, percentage of lipid deposition, and serum lipid levels of TG, TC and LDL-C compared to the Sh-Ctrl group. These data might illustrate that the attenuation of Olfr2 expression may contribute to prevent atherosclerotic lesions. Additionally, when Olfr2 expression was decreased, the therapeutic effects of Corilagin on pathological changes, lipid deposition percentages, and serum lipid levels were diminished (**Figures 4A, 4D**). These outcomes indicated Olfr2 might serve as a critical pathway for Corilagin’s atherosclerosis prevention effects.

### Effects of corilagin on Olfr2 and downstream molecules after downregulating Olfr2 *in vivo* and vitro

**Figure 5:**
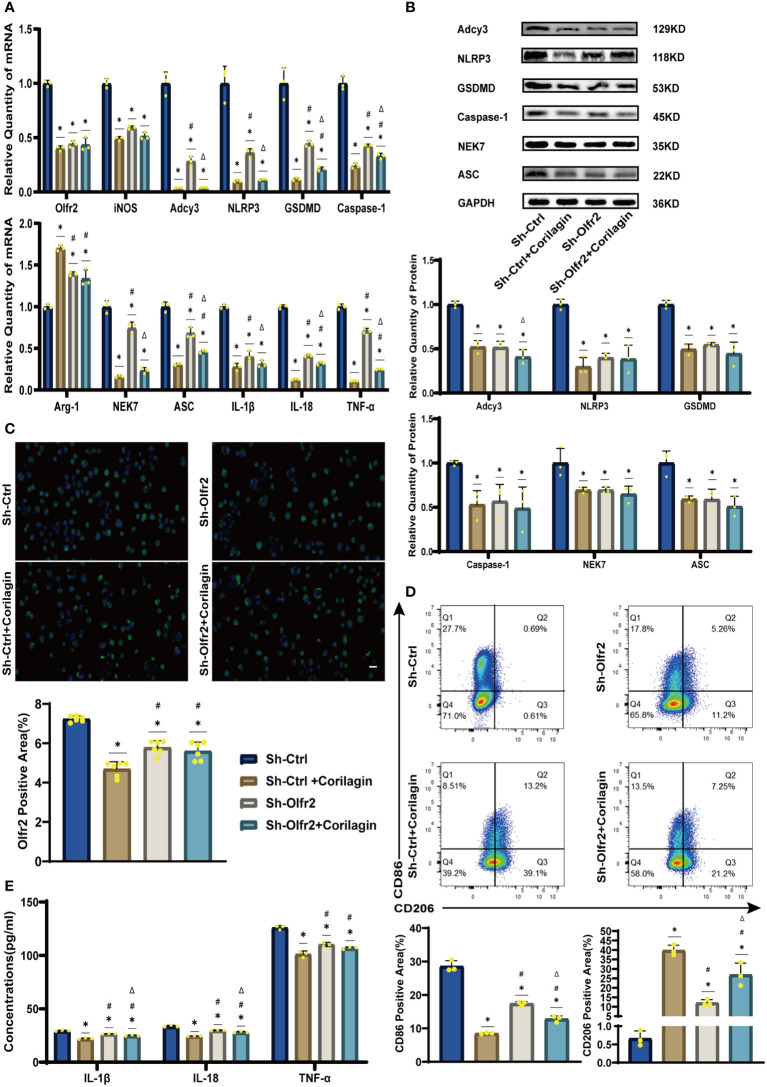
Effects of Corilagin on Olfr2 signal pathway after downregulating Olfr2 *in vitro*. **(A)** mRNA expression of Olfr2, Adcy3, NLRP3, Caspase-1, ASC, NEK7, GSDMD, IL-1β, IL-18, Arg-1, iNOS, and TNF-α in Ana-1 cells was measured by RT-qPCR. ^*^p < 0.05 compared with the Sh-Ctrl group determined by one-way ANOVA test (n = 3), ^#^*p* < 0.05 compared with the Sh-Ctrl+Corilagin group determined by one-way ANOVA test (n = 3), ^Δ^*p* < 0.05 compared with the Sh-Olfr2 group determined by Student’s t-test (n = 3). Data was presented as the mean ± SD. **(B)** Protein expression of Adcy3, NLRP3, GSDMD, Caspase-1, NEK7, and ASC in Ana-1 cells was measured by WB and quantitative analyses of protein level based on WB. ^*^p < 0.05 compared with the Sh-Ctrl group determined by one-way ANOVA test (n = 3), ^#^*p* < 0.05 compared with the Sh-Ctrl+Corilagin group determined by one-way ANOVA test (n = 3), ^Δ^*p* < 0.05 compared with the Sh-Olfr2 group determined by Student’s t-test (n = 3). Data was presented as the mean ± SD. **(C)** Olfr2 expression in Ana-1 cells was visualized by IF and quantitative analyses of Olfr2 level based on IF. ^*^p < 0.05 compared with the Sh-Ctrl group determined by one-way ANOVA test (n = 6), ^#^*p* < 0.05 compared with the Sh-Ctrl+Corilagin group determined by one-way ANOVA test (n = 6), ^Δ^*p* < 0.05 compared with the Sh-Olfr2 group determined by Student’s t-test (n = 6). Data was presented as the mean ± SD. Scale bars, 20μm. **(D)** CD86 and CD206 expression in Ana-1 cells were measured by FC and quantitative analyses of CD86 and CD206 level based on FC. ^*^p < 0.05 compared with the Sh-Ctrl group determined by one-way ANOVA test (n = 3), ^#^*p* < 0.05 compared with the Sh-Ctrl+Corilagin group determined by one-way ANOVA test (n = 3), ^Δ^*p* < 0.05 compared with the Sh-Olfr2 group determined by Student’s t-test (n = 3). Data was presented as the mean ± SD. **(E)** IL-1β, IL-18, and TNF-α in Ana-1 cell supernatant were measured by ELISA. ^*^p < 0.05 compared with the Sh-Ctrl group determined by one-way ANOVA test (n = 3), ^#^*p* < 0.05 compared with the Sh-Ctrl+Corilagin group determined by one-way ANOVA test (n = 3), ^Δ^*p* < 0.05 compared with the Sh-Olfr2 group determined by Student’s t-test (n = 3). Data was presented as the mean ± SD.

To validate our hypothesis that Corilagin could suppress NLRP3 inflammasome activation and subsequent cytokine production, macrophage polarization, and pyroptosis by inhibiting the Olfr2 signaling pathway, we investigated the changes of Olfr2 and downstream molecules expression after downregulating Olfr2. Compared to the Sh-Ctrl group, Olfr2 mRNA levels in the Sh-Olfr2 group fell nearly 50% and 60% in the ApoE^−/−^ mouse and Ana-1 cell models (**Figures 4B, 4A**). These results confirmed the effective inhibition of Olfr2 expression in this study. As illustrated in **Figures 4A–C, 4E**, **5A–5E**, the reduction in Olfr2, iNOS, Adcy3, NLRP3, GSDMD, Caspase-1, NEK7, ASC, IL-1β, IL-18, TNF-α, and CD86 induced by Corilagin was less pronounced when Olfr2 expression was decreased. Therefore, we inferred that Corilagin could suppress NLRP3 inflammasome activation to prevent inflammatory cytokines secretion, markers of M1 macrophage polarization expression, and pyroptosis-related molecule production via inhibiting the Olfr2 signaling pathway.

### Effects of corilagin on atherosclerosis by upregulating Olfr2 *in vivo*

**Figure 6:**
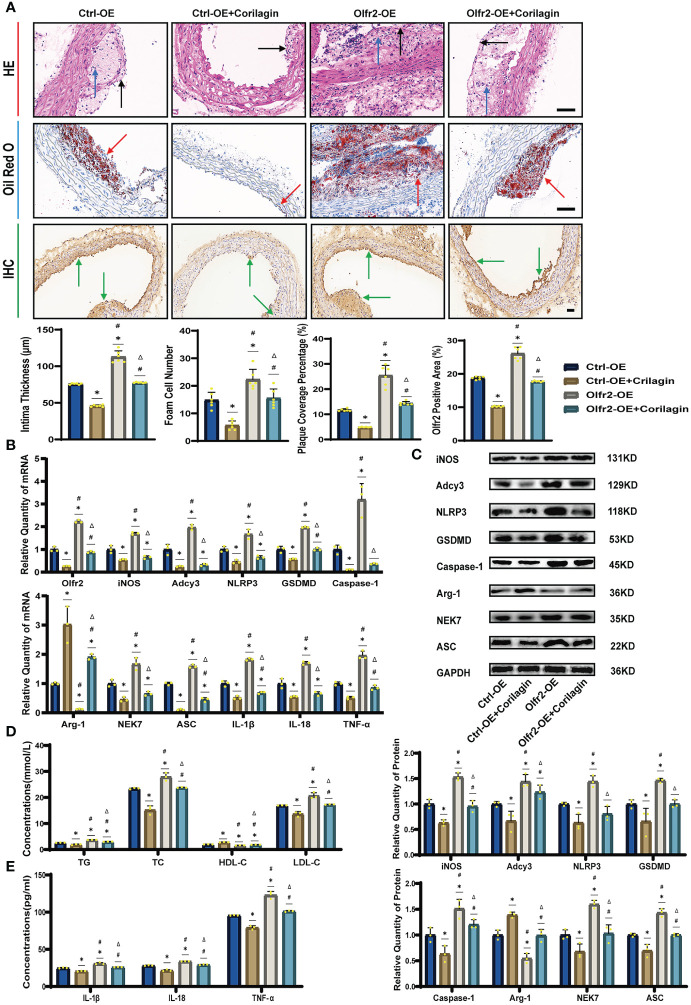
Effects of Corilagin on atherosclerosis and Olfr2 signal pathway after upregulating Olfr2 *in vivo*. **(A)** Pathological changes, plaque coverage percentage, and Olfr2 expression in mouse aorta were visualized by HE, Oil red O, and IHC staining respectively and quantitative analyses of intima thickness and foam cell number based on HE, plaque coverage percentage based on Oil red O, and Olfr2 expression based on IHC. Black arrows indicated aorta intima and blue arrows indicated foam cell and fibrous tissue in HE. Yellow arrows indicated lipid plaque in Oil red O. Green arrows indicated the area of Olfr2 expression in IHC. ^*^p < 0.05 compared with the Ctrl-OE group determined by one-way ANOVA test (n = 6), ^#^*p* < 0.05 compared with the Ctrl-OE+Corilagin group determined by one-way ANOVA test (n = 6), ^Δ^*p* < 0.05 compared with the Ctrl-Olfr2 group determined by Student’s t test (n = 6). Data was presented as the mean ± SD. Scale bars, 50μm. **(B)** mRNA expression of Olfr2, Adcy3, NLRP3, Caspase-1, ASC, NEK7, GSDMD, IL-1β, IL-18, Arg-1, iNOS, and TNF-α in mouse aorta was measured by RT-qPCR. ^*^p < 0.05 compared with the Ctrl-OE group determined by one-way ANOVA test (n = 3), ^#^*p* < 0.05 compared with the Ctrl-OE+Corilagin group determined by one-way ANOVA test (n = 3), ^Δ^*p* < 0.05 compared with the Ctrl-Olfr2 group determined by Student’s t test (n = 3). Data was presented as the mean ± SD. **(C)** Protein expression of iNOS, Adcy3, NLRP3, GSDMD, Caspase-1, NEK7, Arg-1, and ASC in mouse aorta was measured by WB and quantitative analyses of protein level based on WB. ^*^p < 0.05 compared with the Ctrl-OE group determined by one-way ANOVA test (n = 3), ^#^*p* < 0.05 compared with the Ctrl-OE+Corilagin group determined by one-way ANOVA test (n = 3), ^Δ^*p* < 0.05 compared with the Ctrl-Olfr2 group determined by Student’s t test (n = 3). Data was presented as the mean ± SD. **(D)** Serum lipids of mice were detected by an automatic biochemical analyzer. WB and quantitative analyses of protein level based on WB. ^*^p < 0.05 compared with the Ctrl-OE group determined by one-way ANOVA test (n = 3), ^#^*p* < 0.05 compared with the Ctrl-OE+Corilagin group determined by one-way ANOVA test (n = 3), ^Δ^*p* < 0.05 compared with the Ctrl-Olfr2 group determined by Student’s t test (n = 3). Data was presented as the mean ± SD. **(E)** IL-1β, IL-18, and TNF-α in the mouse serum were measured by ELISA. ^*^p < 0.05 compared with the Ctrl-OE group determined by one-way ANOVA test (n = 3), ^#^*p* < 0.05 compared with the Ctrl-OE+Corilagin group determined by one-way ANOVA test (n = 3), ^Δ^*p* < 0.05 compared with the Ctrl-Olfr2 group determined by Student’s t test (n = 3). Data was presented as the mean ± SD.

To verify that Corilagin exerted anti-atherosclerotic effects via the Olfr2 signaling pathway, we conducted *in vivo* experiments involving the upregulation of Olfr2 to monitor changes in atherosclerotic conditions. As depicted in **Figures 6A, 6D**, the Olfr2-OE group exhibited more severe pathological changes, increased lipid deposition percentage, and elevated serum lipid levels of TG, TC and LDL-C compared to the Ctrl-OE group. These findings suggested that boosting Olfr2 levels could intensify atherosclerosis. Moreover, when Olfr2 expression was augmented, the therapeutic effects of Corilagin on reducing histological changes and lipid deposition were more pronounced (**Figures 6A, 6D**). These results indicated that Corilagin’s influence on atherosclerosis was indeed associated with Olfr2 expression.

### Effects of corilagin on Olfr2 and downstream molecules after upregulating Olfr2 *in vivo* and vitro

**Figure 7:**
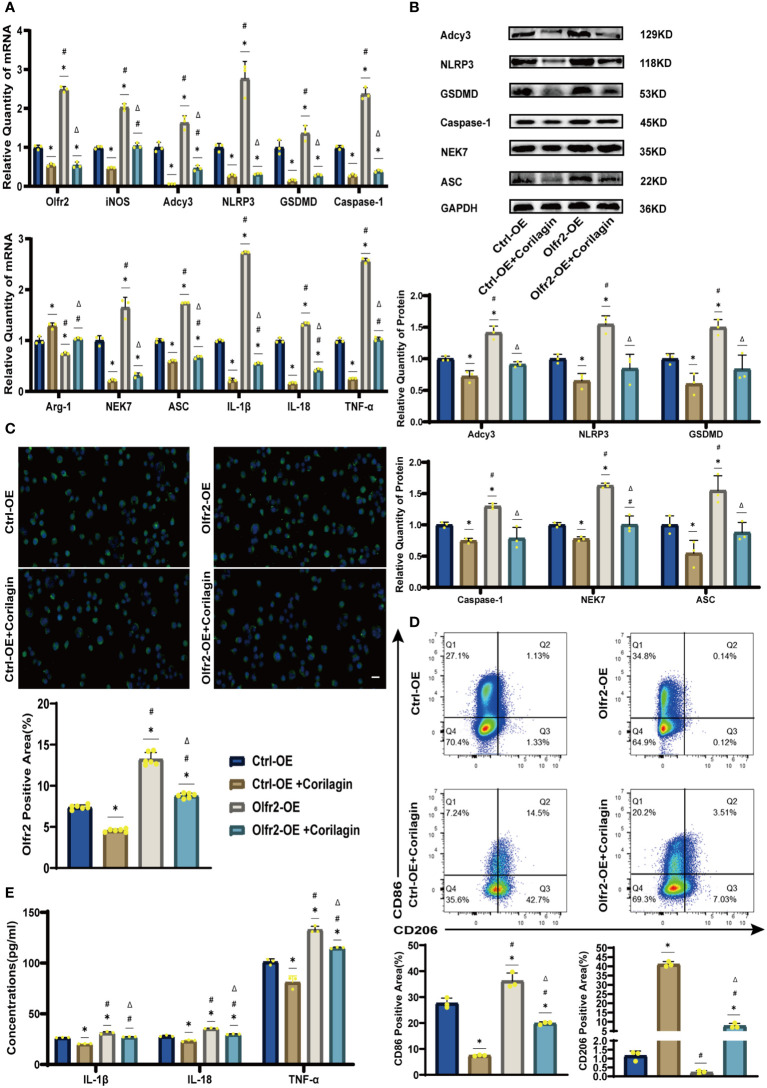
Effects of Corilagin on Olfr2 signal pathway after upregulating Olfr2 *in vitro* **(A)** mRNA expression of Olfr2, Adcy3, NLRP3, Caspase-1, ASC, NEK7, GSDMD, IL-1β, IL-18, Arg-1, iNOS, and TNF-α in Ana-1 cells was measured by RT-qPCR. ^*^p < 0.05 compared with the Ctrl-OE group determined by one-way ANOVA test (n = 3), ^#^*p* < 0.05 compared with the Ctrl-OE+Corilagin group determined by one-way ANOVA test (n = 3), ^Δ^*p* < 0.05 compared with the Ctrl-Olfr2 group determined by Student’s t test (n = 3). Data was presented as the mean ± SD. **(B)** Protein expression of Adcy3, NLRP3, GSDMD, Caspase-1, NEK7, and ASC in Ana-1 cells was measured by WB and quantitative analyses of protein level based on WB. ^*^p < 0.05 compared with the Ctrl-OE group determined by one-way ANOVA test (n = 3), ^#^*p* < 0.05 compared with the Ctrl-OE+Corilagin group determined by one-way ANOVA test (n = 3), ^Δ^*p* < 0.05 compared with the Ctrl-Olfr2 group determined by Student’s t test (n = 3). Data was presented as the mean ± SD. **(C)** Olfr2 expression in Ana-1 cells was visualized by IF and quantitative analyses of Olfr2 level based on IF. ^*^p < 0.05 compared with the Ctrl-OE group determined by one-way ANOVA test (n = 6), ^#^*p* < 0.05 compared with the Ctrl-OE+Corilagin group determined by one-way ANOVA test (n = 6), ^Δ^*p* < 0.05 compared with the Ctrl-Olfr2 group determined by Student’s t test (n = 6). Data was presented as the mean ± SD. Scale bars, 20μm. **(D)** CD86 and CD206 expression in Ana-1 cells were measured by FC and quantitative analyses of CD86 and CD206 level based on FC. ^*^p < 0.05 compared with the Ctrl-OE group determined by one-way ANOVA test (n = 3), ^#^*p* < 0.05 compared with the Ctrl-OE+Corilagin group determined by one-way ANOVA test (n = 3), ^Δ^*p* < 0.05 compared with the Ctrl-Olfr2 group determined by Student’s t test (n = 3). Data was presented as the mean ± SD. **(E)** IL-1β, IL-18, and TNF-α in Ana-1 cell supernatant were measured by ELISA. ^*^p < 0.05 compared with the Ctrl-OE group determined by one-way ANOVA test (n = 3), ^#^*p* < 0.05 compared with the Ctrl-OE+Corilagin group determined by one-way ANOVA test (n = 3), ^Δ^*p* < 0.05 compared with the Ctrl-Olfr2 group determined by Student’s t test (n = 3). Data was presented as the mean ± SD.

We augmented Olfr2 expression in ApoE^−/−^ mice and Ana-1 cell models to further elucidate the molecular mechanisms of Corilagin anti-atherosclerotic effects. Compared to the Ctrl-OE group, the level of Olfr2 mRNA in the Olfr2-OE group increased by approximately 120% and 150% *in vivo* and vitro samples, respectively (**Figure 6B**, **7A**). This elevation indicates a successful increase in Olfr2 expression following upregulation. Notably, with enhanced Olfr2 expression, Corilagin more effectively reduced the expression of molecules involved in the Olfr2 signaling pathway in the Olfr2-OE+Corilagin group (**Figures 6A–6C, 6E**, **7A–7E**). This trend supported and potentially strengthened the conclusion illustrated in the second part of results, affirming the role of Corilagin in modulating the Olfr2 signaling pathway.

## Discussion

Atherosclerosis and its associated complications represent significant sources of morbidity and mortality, making the development of effective therapies for both prevention and treatment crucial (1–4). Although a variety of pharmaceutical options are available for managing atherosclerotic disease, limitations persist, such as resistance or intolerance to statins (43) and increased bleeding risk associated with antiplatelet medications (44). Concurrently, Chinese herbal medicines are often regarded as complementary or alternative treatment for atherosclerosis (45).

Inflammation is a critical driver of atherosclerosis. Thus, targeting therapeutic interventions at inflammatory pathways is essential for improving atherosclerotic outcomes (2). Several signaling pathways implicated in the inflammatory response are associated with atherosclerosis, including the NLRP3 inflammasome, TLRs, proprotein convertase subtilisin/kexin type 9, Notch, and Wnt signaling pathways. Notably, the NLRP3 inflammasome pathway has emerged as a prominent target for atherosclerotic disease treatment (2, 8, 46). In conjunction with TLR4, Olfr2 expressed in mouse vascular macrophages can active NLRP3 inflammasome to promote the development of atherosclerosis (35). According to previous findings, Corilagin can inhibit NLRP3 inflammasome activation and pyroptosis in ischemia-reperfusion induced intestinal and lung injury (38), and ameliorate atherosclerosis by restraining the TLR4 signaling pathway (17, 40). Consequently, this study aims to explore whether Corilagin can influence atherosclerosis through the Olfr2 signaling pathway.

After administering Corilagin to ApoE^−/−^ mice on a high-fat and cholesterol diet, we observed a reduction in serum lipid levels of TG, TC, and LDL-C, alongside alleviated pathological changes and lipid deposition. These results suggest a pronounced therapeutic effect of Corilagin on atherosclerosis. In both the mouse aorta and cellular models, expressions of the Olfr2 signaling pathway related molecules (Olfr2 and Adcy3) and NLRP3 inflammasome associated effectors (NLRP3, Caspase-1, NEK7 and ASC), were diminished following Corilagin treatment. Additionally, inflammatory factors (IL-1β, IL-18, and TNF-α), M1 macrophage polarization markers (iNOS and CD86), and the pyroptosis-related molecule (GSDMD), were also reduced. In conclusion, we hypothesized that Corilagin mitigated atherosclerotic development by suppressing NLRP3 inflammasome activation, thereby curtailing the expression of inflammatory cytokines, M1 macrophage polarization, and pyroptosis through the inhibition of the Olfr2 signaling pathway.

To further validate the effect of Corilagin, we modulated Olfr2 expression both in Ana-1 cells and in animal models, involving both upregulation and downregulation. According to Orecchioni et al., enhancing Olfr2 levels can intensify atherosclerosis, and genetic manipulation to reduce Olfr2 in mice has shown a significant decrease in atherosclerotic plaques (35). In our experiments that modulated Olfr2 expression both *in vitro* and *in vivo*, we observed analogous outcomes confirming that the Olfr2 signaling pathway indeed activated the NLRP3 inflammasome, exacerbating atherosclerosis development and progression. Upon downregulation of Olfr2, no substantial differences in LDL-C levels or pathological changes were noted regardless of Corilagin treatment, while lipid plaque percentages were reduced, and the expression levels of Olfr2, iNOS, Adcy3, NLRP3, GSDMD, Caspase-1, NEK7, ASC, IL-1β, IL-18, TNF-α, and CD86 were similar or decreased with Corilagin treatment. Conversely, when Olfr2 expression was increased, the effects of Corilagin in mitigating atherosclerosis and suppressing the Olfr2 signaling pathway were enhanced. Consequently, our findings suggested the anti-atherosclerotic effects of Corilagin might be attributed to its interaction with the Olfr2 signaling pathway. Previous studies have shown that Corilagin can reduce serum lipid levels in atherosclerotic animal models (40, 41). Our study found a same trend and it might be related to the Olfr2 signaling pathway. Corilagin may directly or indirectly affect various lipid regulatory systems, to reduce lipid uptake and lipogenesis in cellular and animal experiments (47–49). Nevertheless, there is a limitation in our research that the mechanisms of Corilagin’s effects on serum lipid levels in atherosclerosis, are warranting exploration remains. Additionally, future research should also consider the interaction between Olfr2 and NLRP3, and whether alternative molecular pathways or additional mechanisms within the Olfr2 pathway might contribute to Corilagin’s atherosclerosis-mitigating effects.

**Figure 8:**
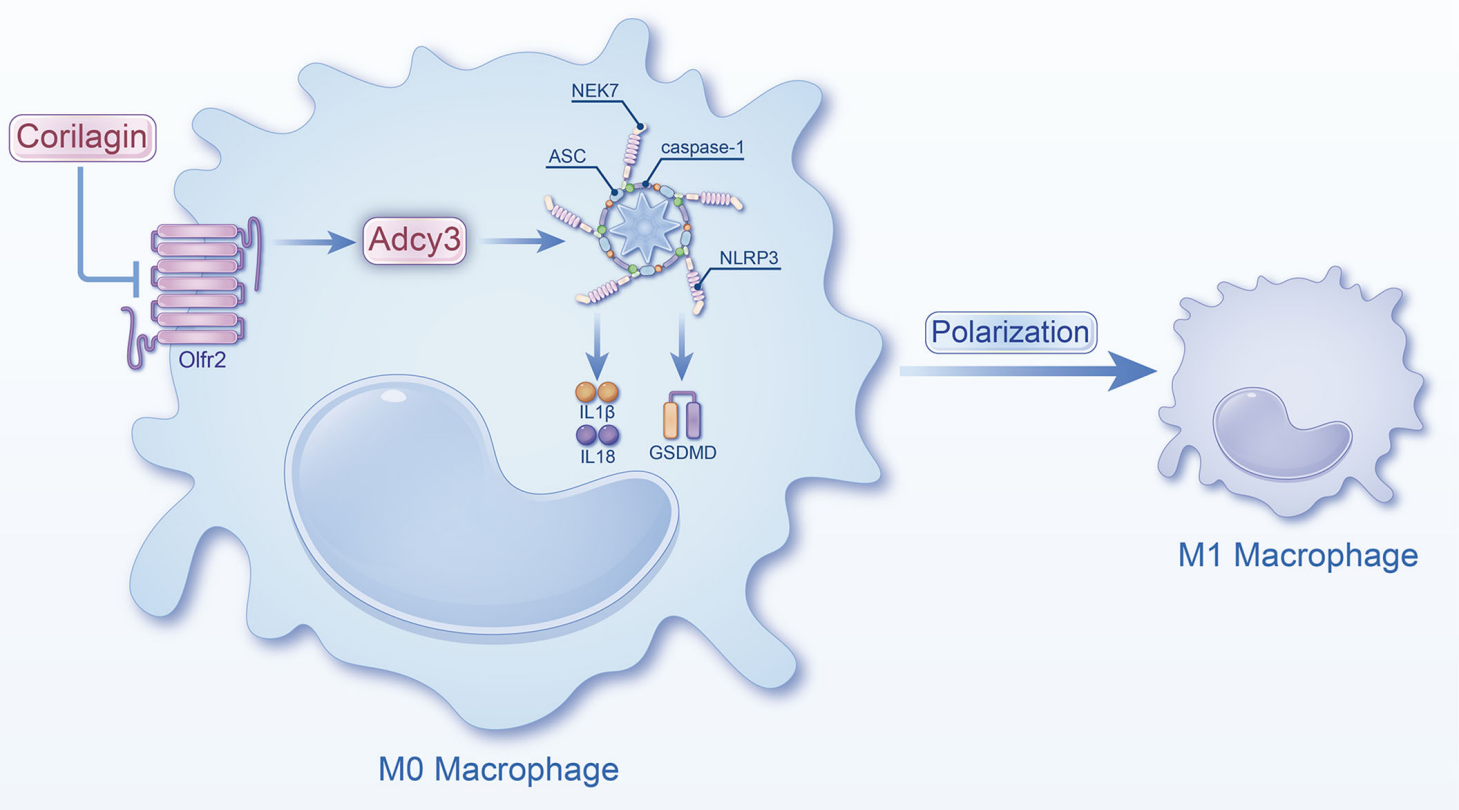
Schematic of the study. Corilagin can inhibit the Olfr2 and Adcy3 expression, then suppress NLRP3 inflammasome activation to prevent inflammatory cytokines expression, M1 macrophage polarization, and cell pyroptosis-related molecule expression.

In summary, Corilagin exhibited therapeutic effects in experimental atherosclerosis models, potentially through the inhibition of the Olfr2 signaling pathway (**Figure 8**). However, a notable limitation of our research was the lack of exploration into whether Corilagin might inhibit downstream molecules through alternative mechanisms.

## Conclusions

Corilagin exerts the anti-atherosclerosis efficacy by inhibiting the Olfr2 signaling pathway *in vitro* and *in vivo*, leading to the suppression of NLRP3 inflammasome activation, reducing inflammatory cytokines expression, M1 macrophage polarization, and expression of pyroptosis-related molecule. This study highlights the promising strategy and significant potential of Corilagin as a treatment for atherosclerosis. Consequently, further investigations into the mechanisms of Corilagin are warranted to identify novel therapeutic approaches.

## Data availability statement

The original contributions presented in the study are included in the article/**Supplementary Material**. Further inquiries can be directed to the corresponding authors.

## Ethics statement

The animal study was approved by Institutional Animal Care and Use Committee of Bainte Biotechnology (Wuhan, China). The study was conducted in accordance with the local legislation and institutional requirements.

## Author contributions

JM: Data curation, Methodology, Writing – original draft. YC: Data curation, Methodology, Writing – original draft. QZ: Investigation, Project administration, Writing – original draft. CL: Software, Writing – original draft. JX: Data curation, Writing – original draft. YW: Formal Analysis, Writing – original draft. DF: Conceptualization, Project administration, Writing – original draft. NH: Conceptualization, Project administration, Writing – original draft. KP: Conceptualization, Project administration, Writing – original draft. EM: Conceptualization, Project administration, Writing – original draft. YL: Conceptualization, Methodology, Project administration, Writing – review & editing. LZ: Conceptualization, Software, Supervision, Writing – review & editing. YD: Data curation, Writing – review & editing.
