# Supplementary material for: Corilagin alleviates atherosclerosis by inhibiting NLRP3 inflammasome activation via the Olfr2 signaling pathway in vitro and in vivo
Source: Front Immunol. 2024 May 13;15:1364161. doi: 10.3389/fimmu.2024.1364161 (PMC11128681; doi:10.3389/fimmu.2024.1364161)
Supplement: Supplementary file 1 [file DataSheet_1.pdf]

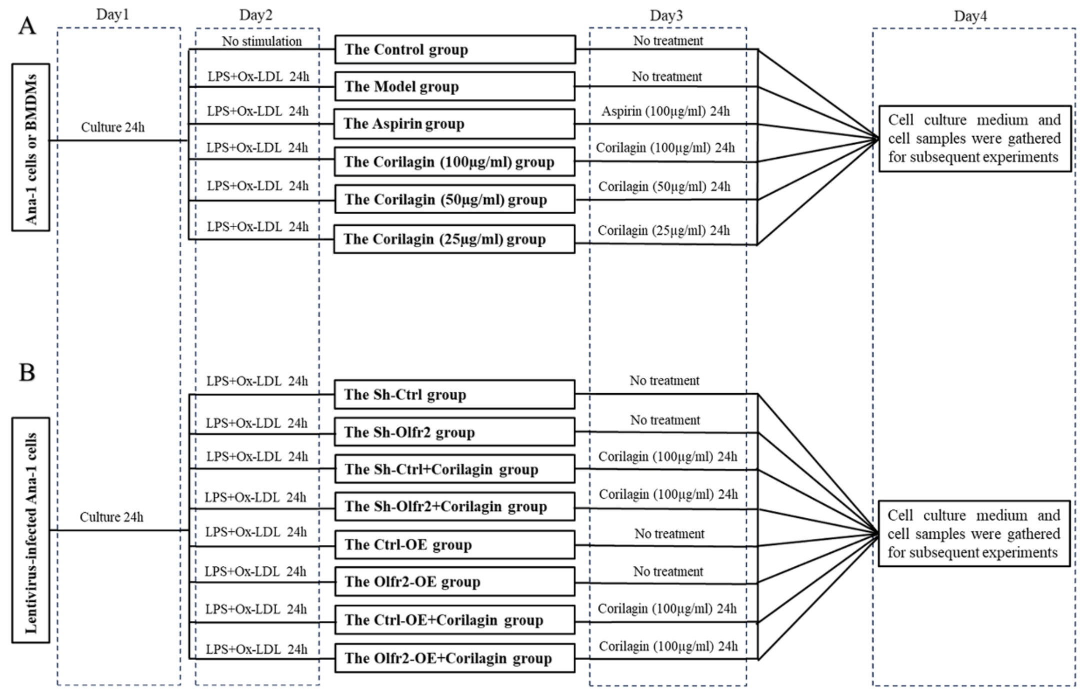

Supplemental figure 1. Flow chart of cell experiments. (A) The process of Ana-1 cells or BMDMs experiment. (B) The process of the lentivirus-infected Ana-1 cells experiment.

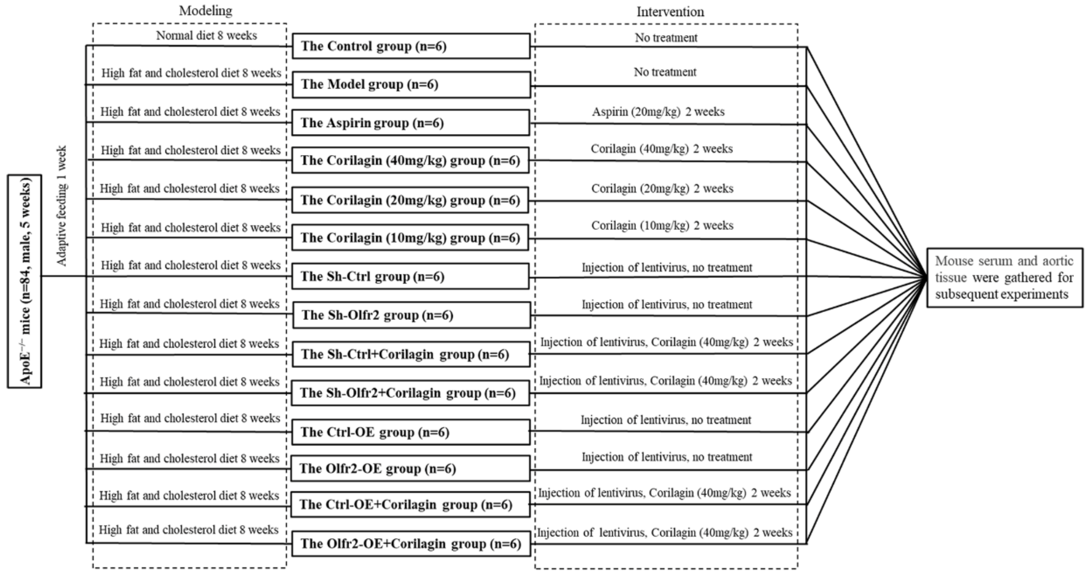

Supplemental figure 2. Flow chart of $ApoE^{-/-}$ mice experiments.

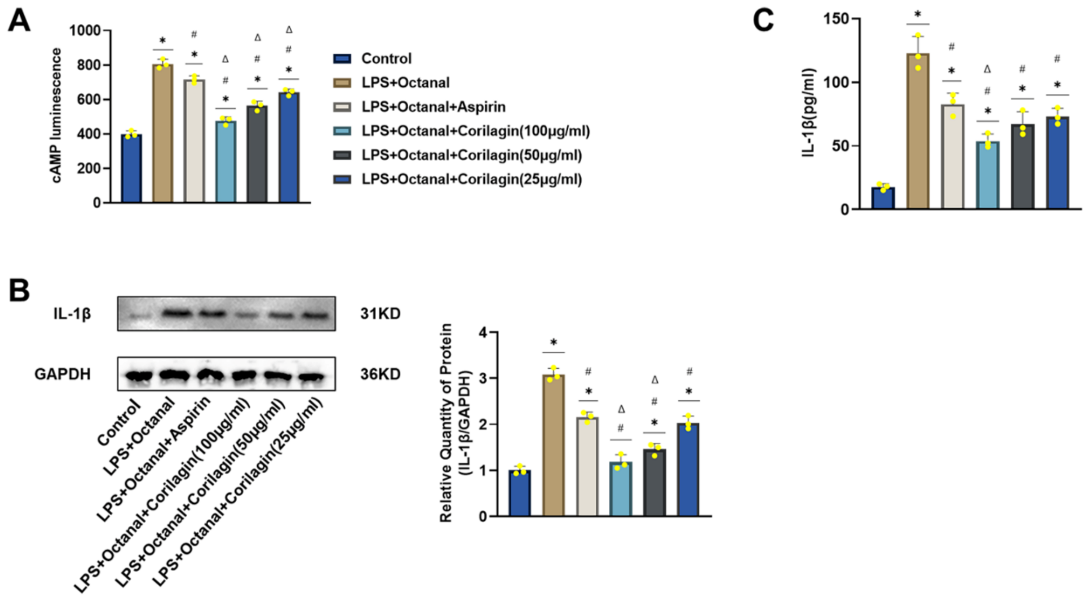

Supplemental figure 3. Corilagin blocks cAMP and IL-1β secretion in Ana-1 cells stimulated by LPS+Octanal. (A) cAMP in Ana-1 cells was assessed by cAMP glow assay. $^{*}p < 0.05$ compared with the Control group, $^{\#}p < 0.05$ compared with the LPS+Octanal group, $^{\Delta}p < 0.05$ compared with the LPS+Octanal+Aspirin group determined by one-way ANOVA test (n = 3). Data was presented as the mean ± SD. (B) Protein expression of IL-1β in Ana-1 cells was measured by WB and quantitative analyses of protein level based on WB. $^{*}p < 0.05$ compared with the Control group, $^{\#}p < 0.05$ compared with the LPS+Octanal group, $^{\Delta}p < 0.05$ compared with the LPS+Octanal+Aspirin group determined by one-way ANOVA test (n = 3). Data was presented as the mean ± SD. (C) IL-1β in Ana-1 cell supernatant was measured by ELISA. $^{*}p < 0.05$ compared with the Control group, $^{\#}p < 0.05$ compared with the LPS+Octanal group, $^{\Delta}p < 0.05$ compared with the LPS+Octanal+Aspirin group determined by one-way ANOVA test (n = 3). Data was presented as the mean ± SD.
