# Supplementary figures and images for: Corilagin alleviates atherosclerosis by inhibiting NLRP3 inflammasome activation via the Olfr2 signaling pathway in vitro and in vivo
Source: Front Immunol. 2024 May 13;15:1364161. doi: 10.3389/fimmu.2024.1364161 (PMC11128681; doi:10.3389/fimmu.2024.1364161)

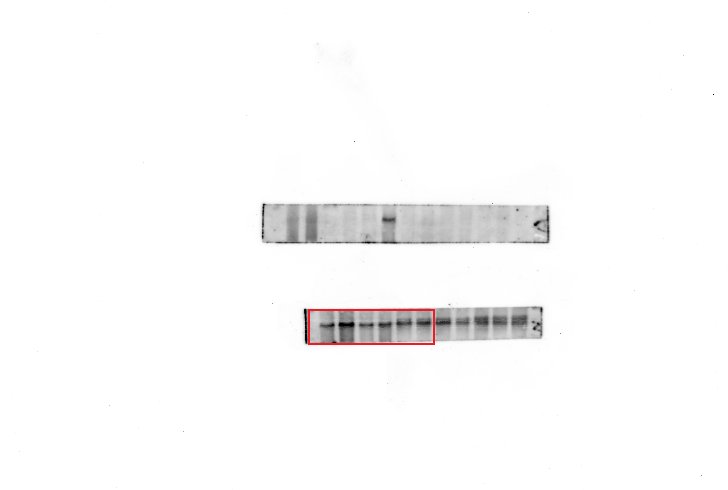

Supplement: Supplementary file 2 [file DataSheet_1.zip › Raw western blot images/Figure 1C/Adcy3.tiff]

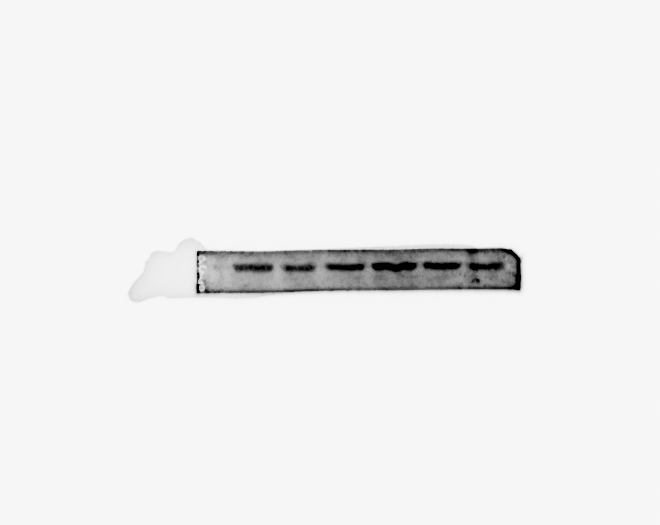

Supplement: Supplementary file 2 [file DataSheet_1.zip › Raw western blot images/Figure 1C/Arg-1.tif]

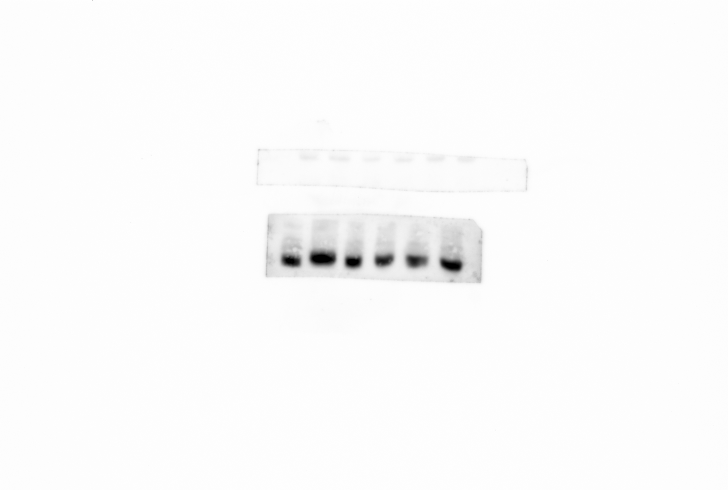

Supplement: Supplementary file 2 [file DataSheet_1.zip › Raw western blot images/Figure 1C/ASC.tif]

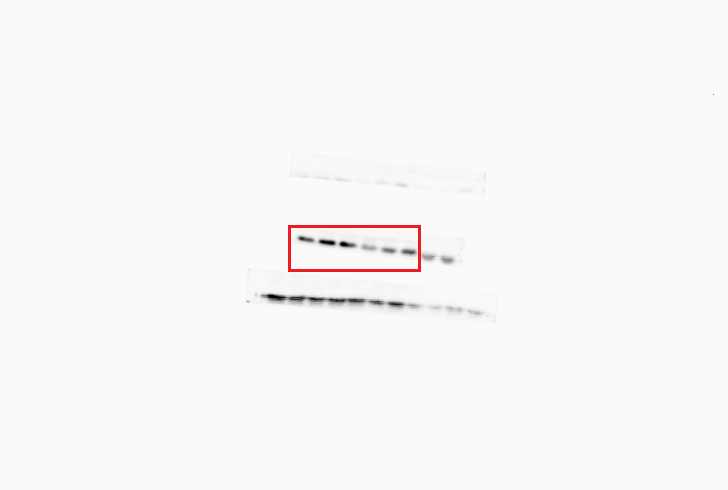

Supplement: Supplementary file 2 [file DataSheet_1.zip › Raw western blot images/Figure 1C/Caspase-1.tiff]

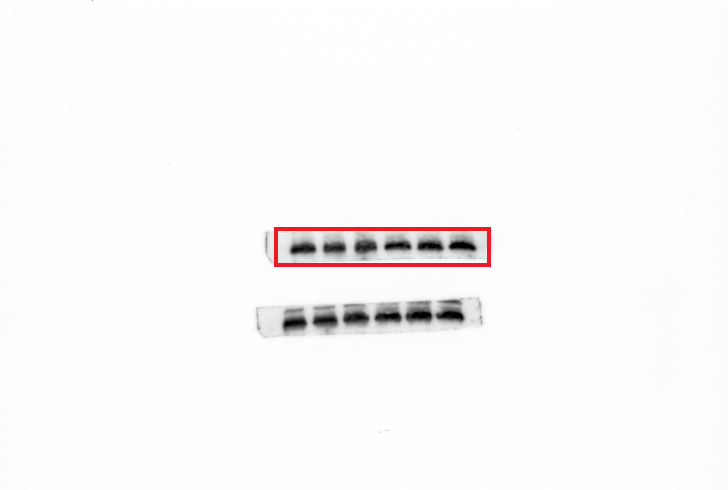

Supplement: Supplementary file 2 [file DataSheet_1.zip › Raw western blot images/Figure 1C/GAPDH.tif]

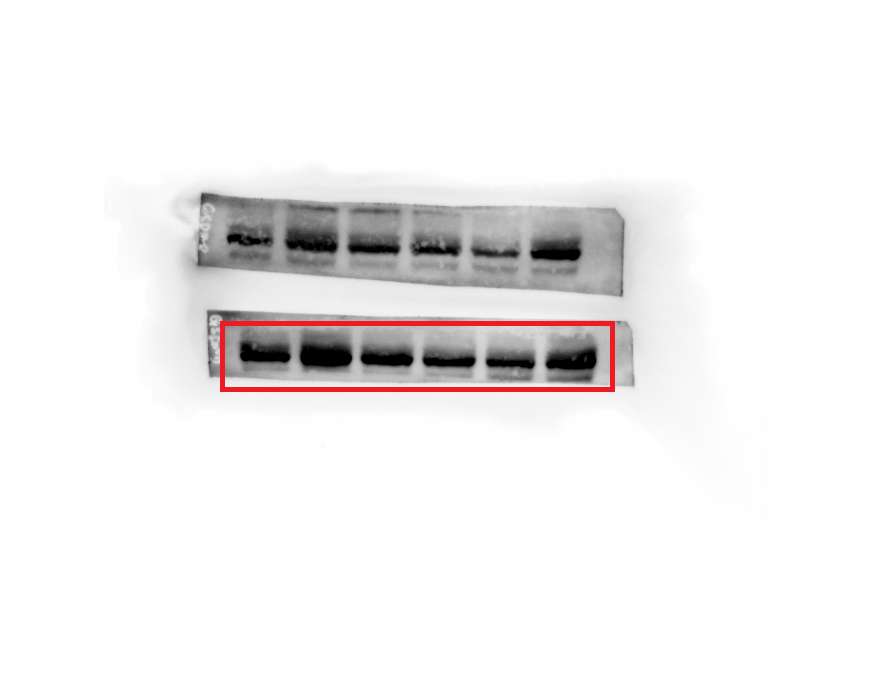

Supplement: Supplementary file 2 [file DataSheet_1.zip › Raw western blot images/Figure 1C/GSDMD.tif]

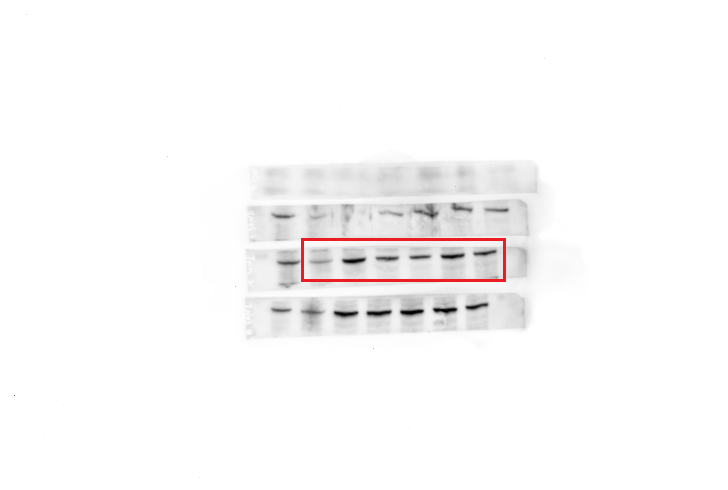

Supplement: Supplementary file 2 [file DataSheet_1.zip › Raw western blot images/Figure 1C/iNOS.tif]

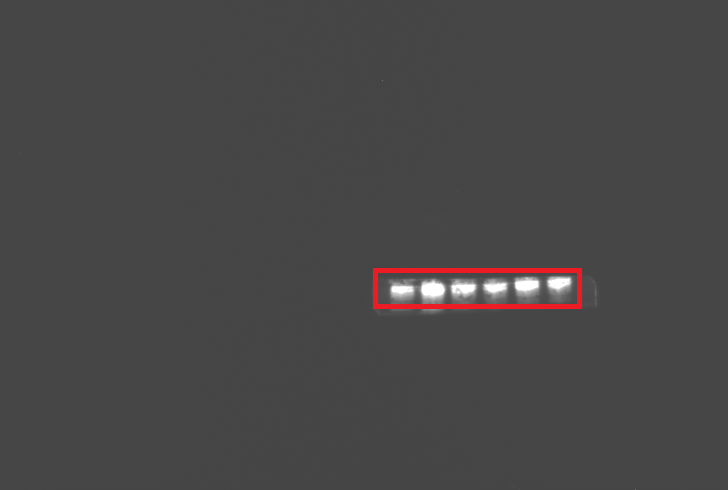

Supplement: Supplementary file 2 [file DataSheet_1.zip › Raw western blot images/Figure 1C/NEK7.tif]

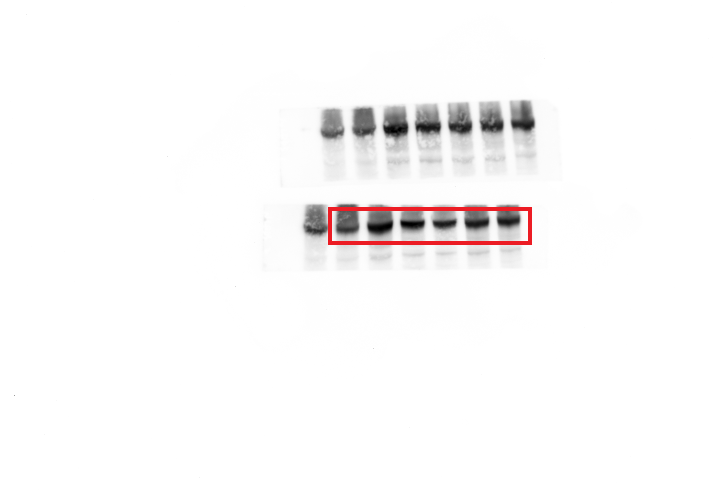

Supplement: Supplementary file 2 [file DataSheet_1.zip › Raw western blot images/Figure 1C/NLRP3.tif]

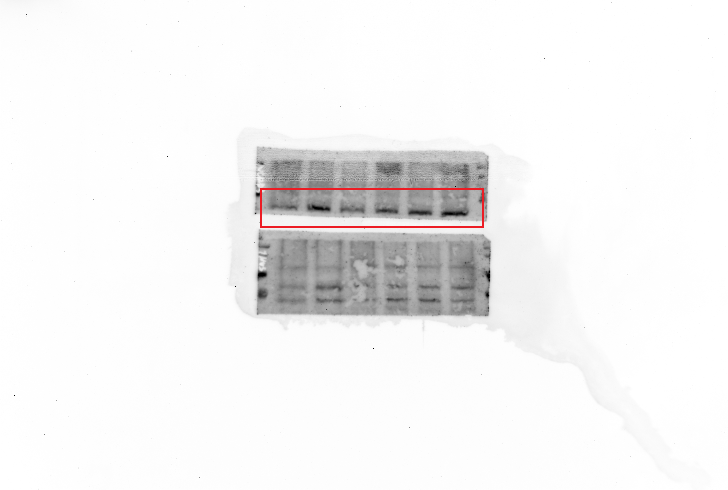

Supplement: Supplementary file 2 [file DataSheet_1.zip › Raw western blot images/Figure 2B/Adcy3.tif]

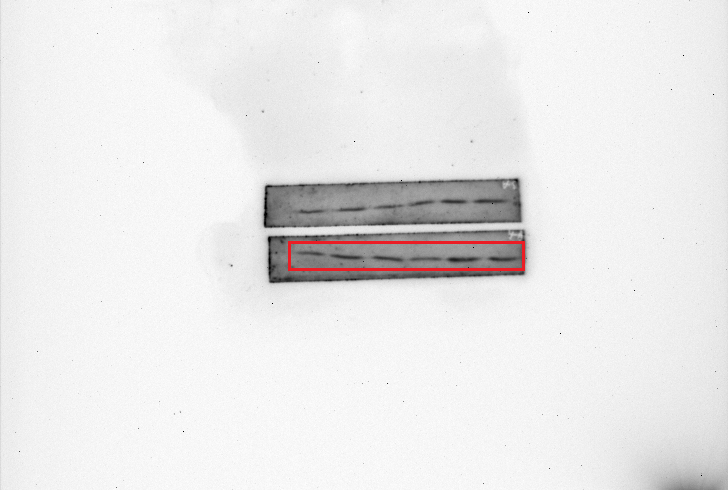

Supplement: Supplementary file 2 [file DataSheet_1.zip › Raw western blot images/Figure 2B/ASC.tif]

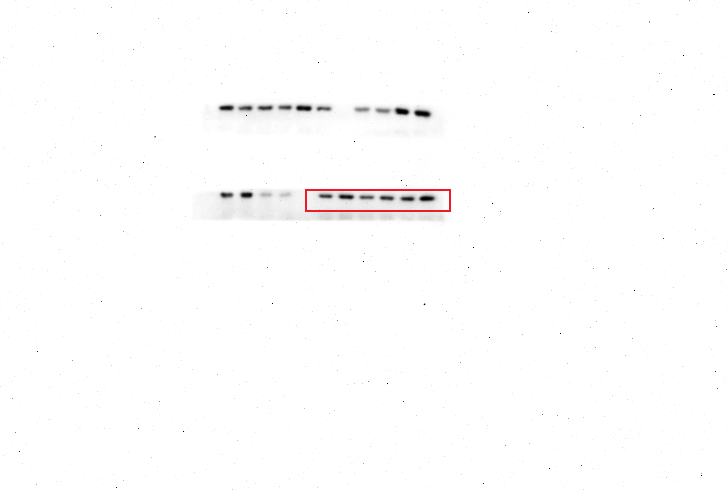

Supplement: Supplementary file 2 [file DataSheet_1.zip › Raw western blot images/Figure 2B/Caspase-1.tiff]

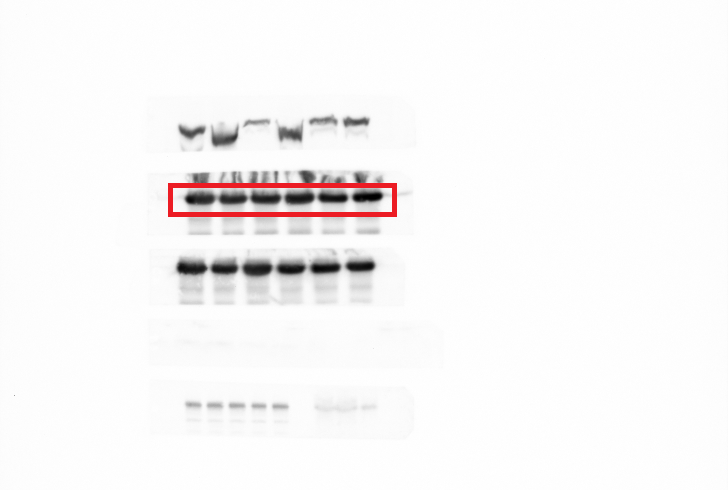

Supplement: Supplementary file 2 [file DataSheet_1.zip › Raw western blot images/Figure 2B/GAPDH.tif]

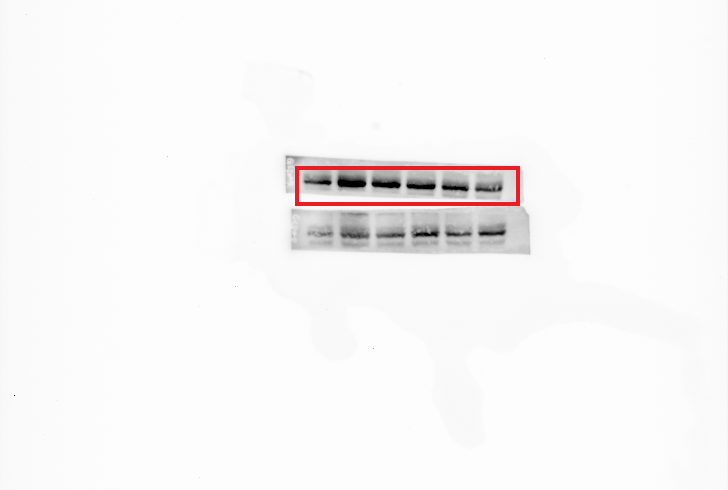

Supplement: Supplementary file 2 [file DataSheet_1.zip › Raw western blot images/Figure 2B/GSDMD.tif]

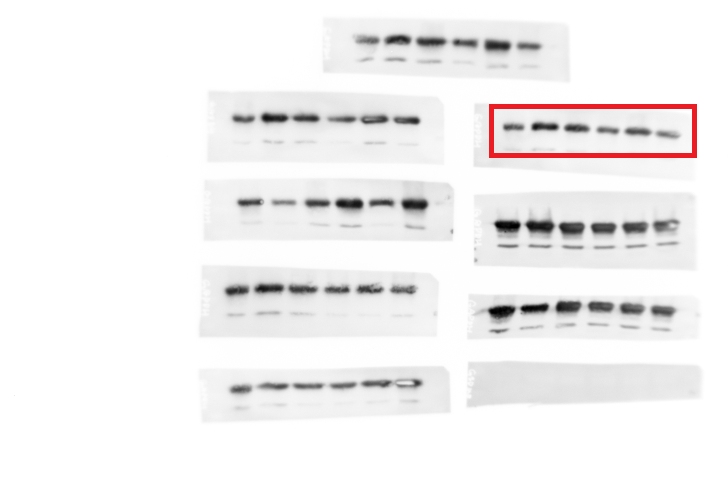

Supplement: Supplementary file 2 [file DataSheet_1.zip › Raw western blot images/Figure 2B/NEK7.tif]

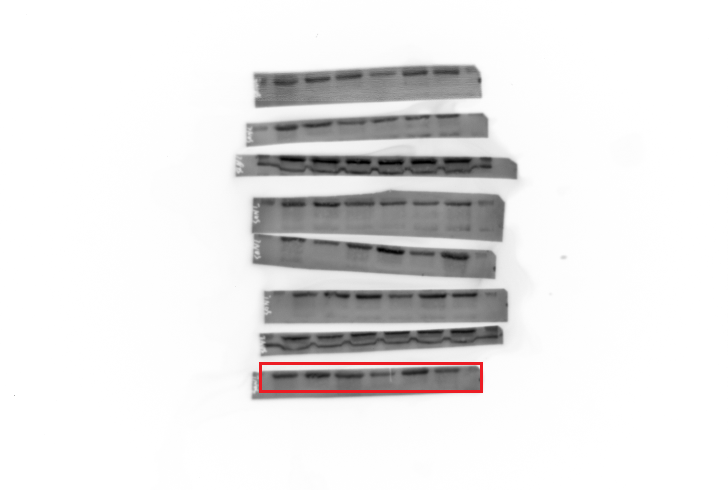

Supplement: Supplementary file 2 [file DataSheet_1.zip › Raw western blot images/Figure 2B/NLRP3.tif]

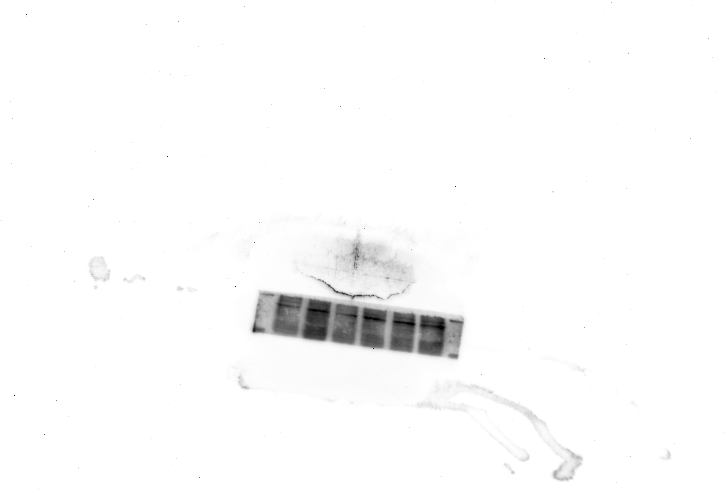

Supplement: Supplementary file 2 [file DataSheet_1.zip › Raw western blot images/Figure 3B/Adcy3.tif]

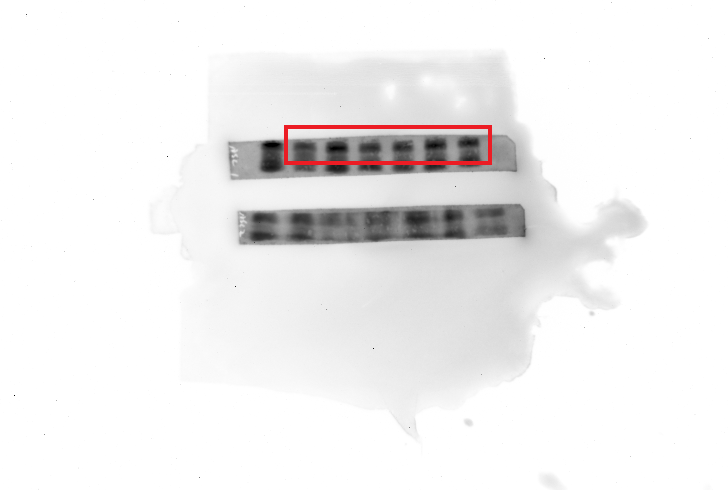

Supplement: Supplementary file 2 [file DataSheet_1.zip › Raw western blot images/Figure 3B/ASC.tif]

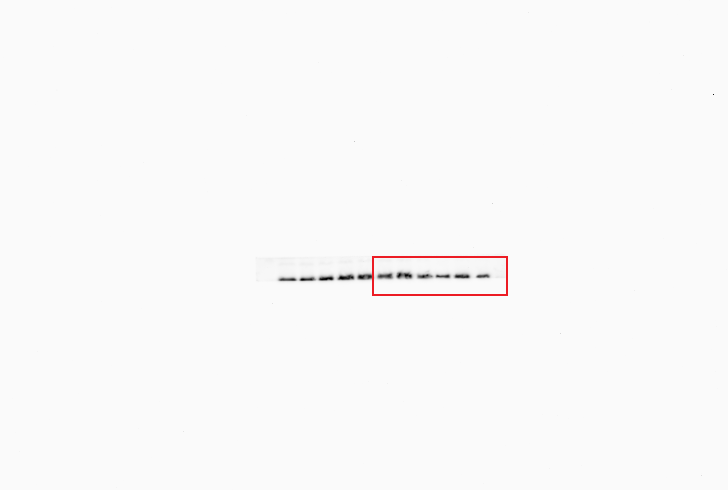

Supplement: Supplementary file 2 [file DataSheet_1.zip › Raw western blot images/Figure 3B/Caspase-1.tiff]

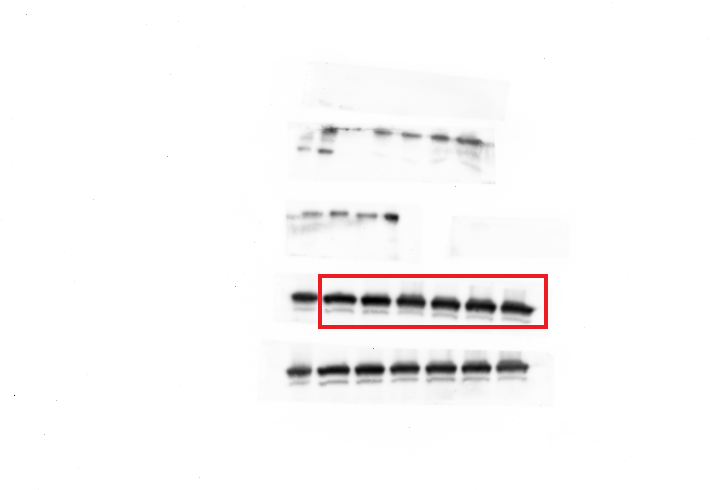

Supplement: Supplementary file 2 [file DataSheet_1.zip › Raw western blot images/Figure 3B/GAPDH.tif]

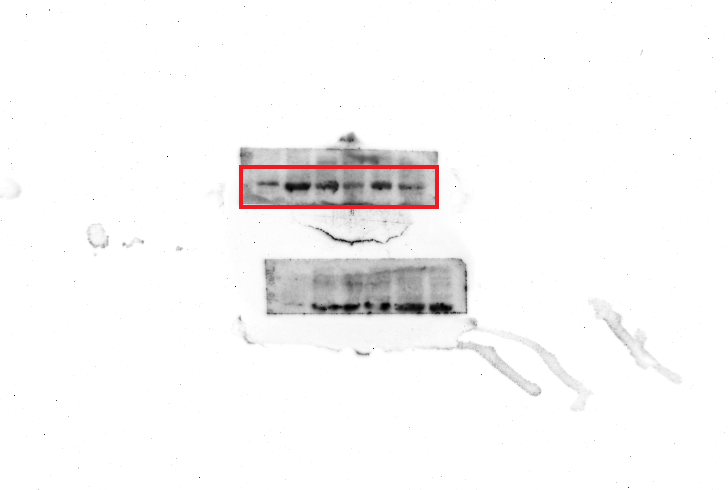

Supplement: Supplementary file 2 [file DataSheet_1.zip › Raw western blot images/Figure 3B/GSDMD.tif]

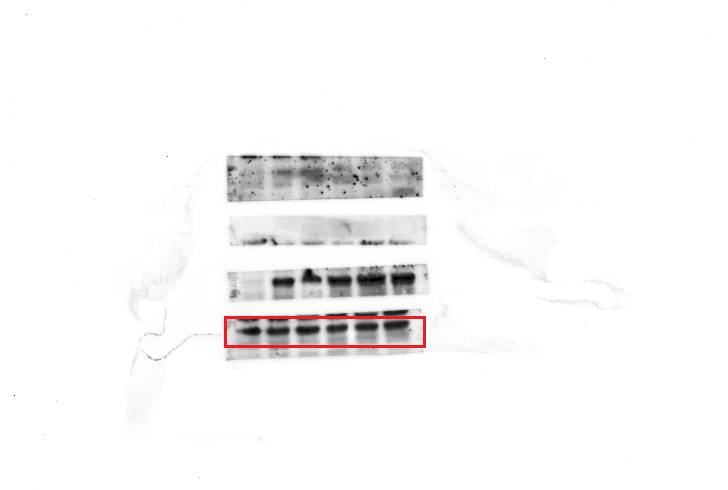

Supplement: Supplementary file 2 [file DataSheet_1.zip › Raw western blot images/Figure 3B/NEK7.tif]

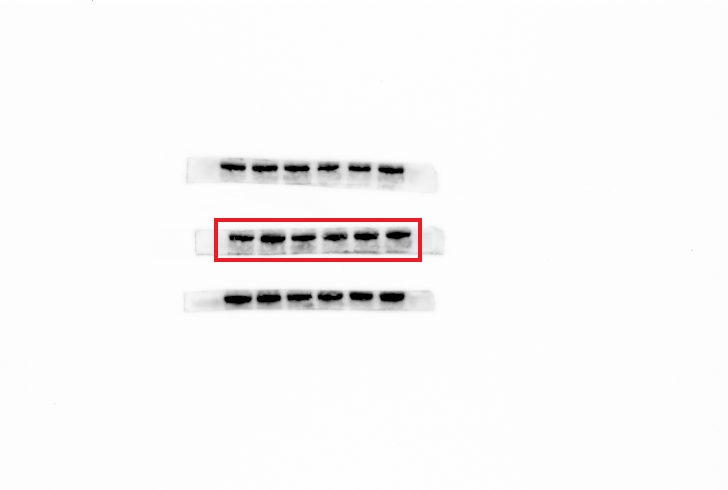

Supplement: Supplementary file 2 [file DataSheet_1.zip › Raw western blot images/Figure 3B/NLRP3.tif]

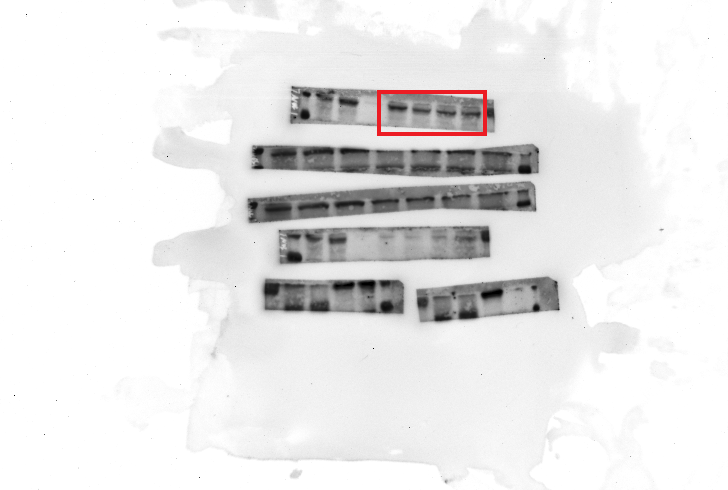

Supplement: Supplementary file 2 [file DataSheet_1.zip › Raw western blot images/Figure 4C/Adcy3.tif]

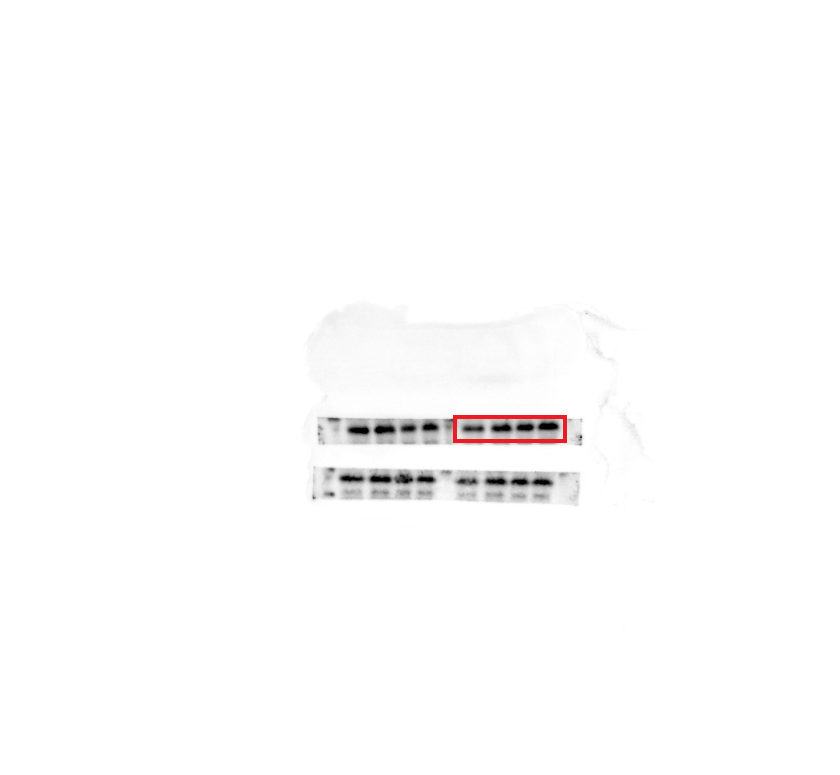

Supplement: Supplementary file 2 [file DataSheet_1.zip › Raw western blot images/Figure 4C/Arg-1.tiff]

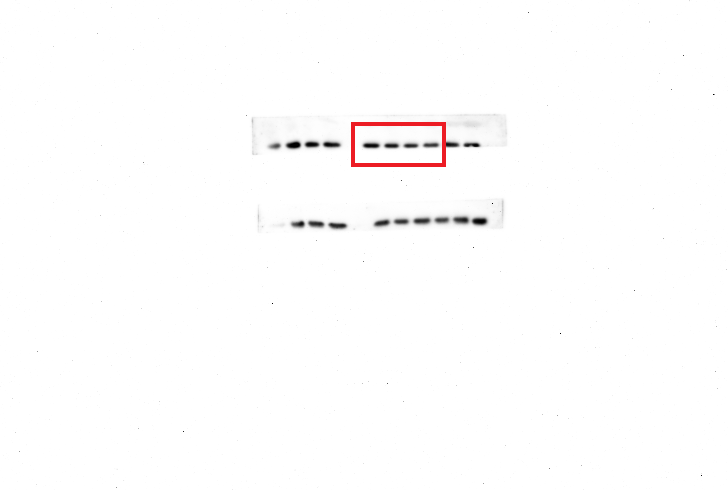

Supplement: Supplementary file 2 [file DataSheet_1.zip › Raw western blot images/Figure 4C/ASC.tiff]

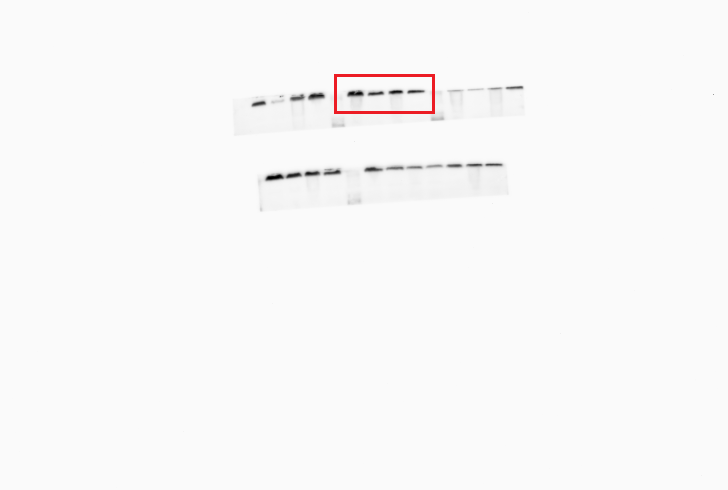

Supplement: Supplementary file 2 [file DataSheet_1.zip › Raw western blot images/Figure 4C/Caspase-1.tiff]

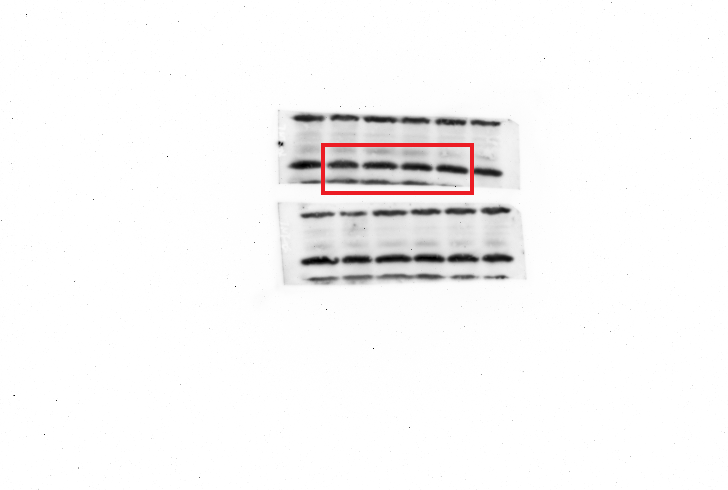

Supplement: Supplementary file 2 [file DataSheet_1.zip › Raw western blot images/Figure 4C/GAPDH.tif]

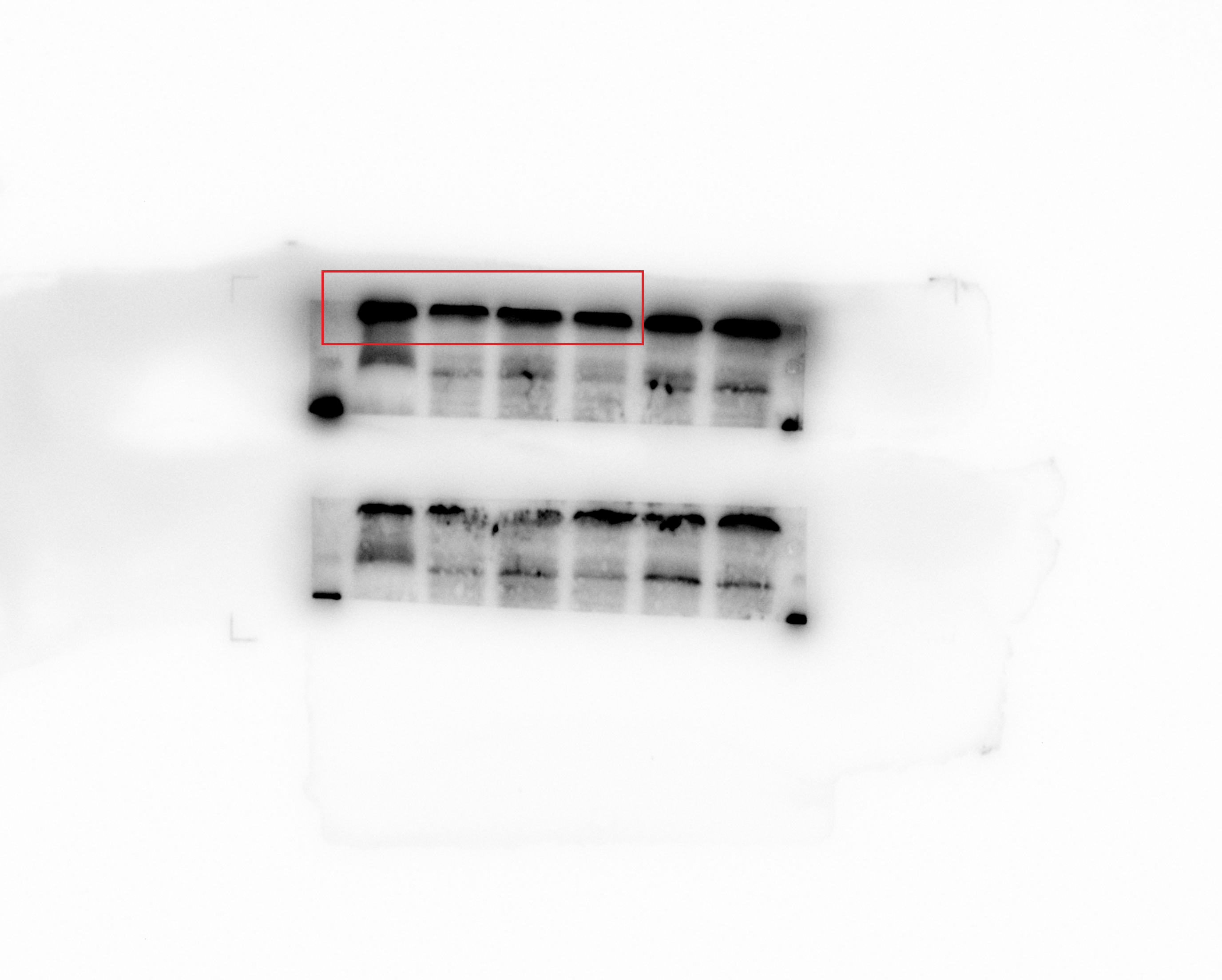

Supplement: Supplementary file 2 [file DataSheet_1.zip › Raw western blot images/Figure 4C/GSDMD.tiff]

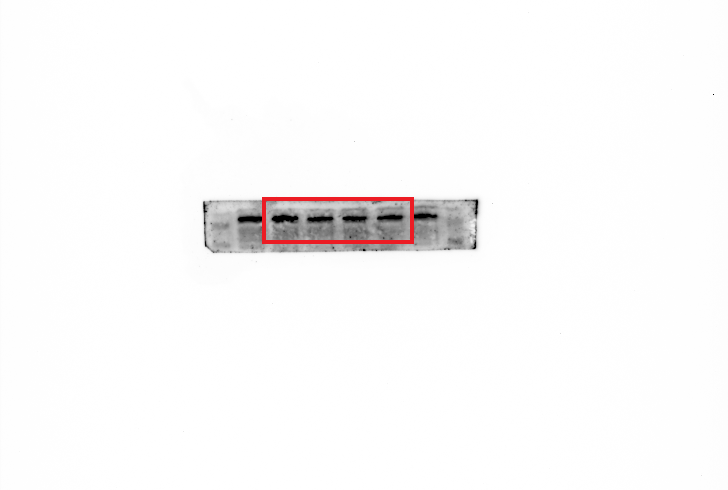

Supplement: Supplementary file 2 [file DataSheet_1.zip › Raw western blot images/Figure 4C/iNOS.tif]

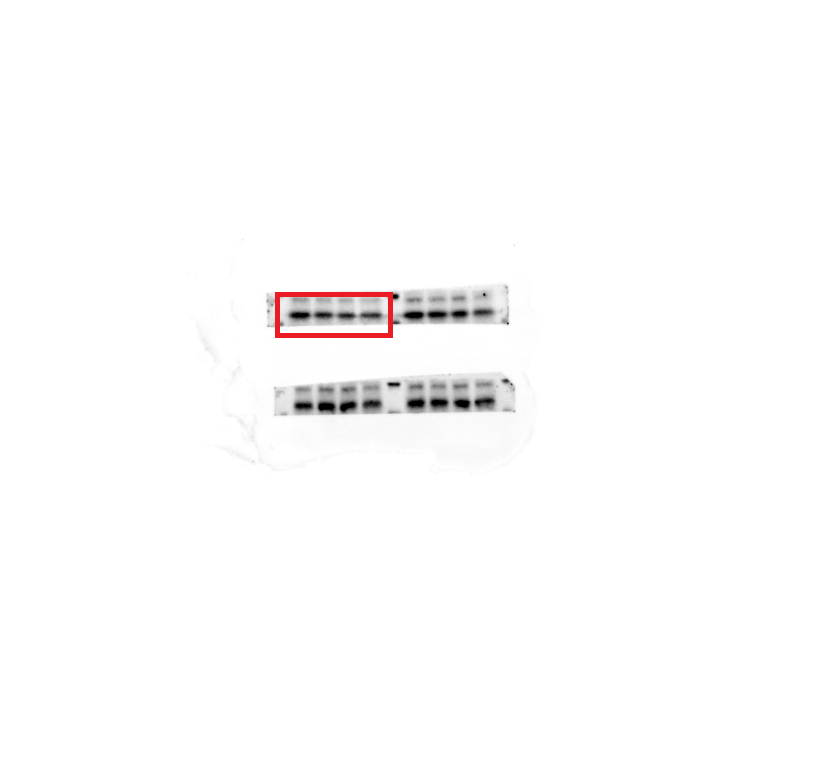

Supplement: Supplementary file 2 [file DataSheet_1.zip › Raw western blot images/Figure 4C/NEK7.tiff]

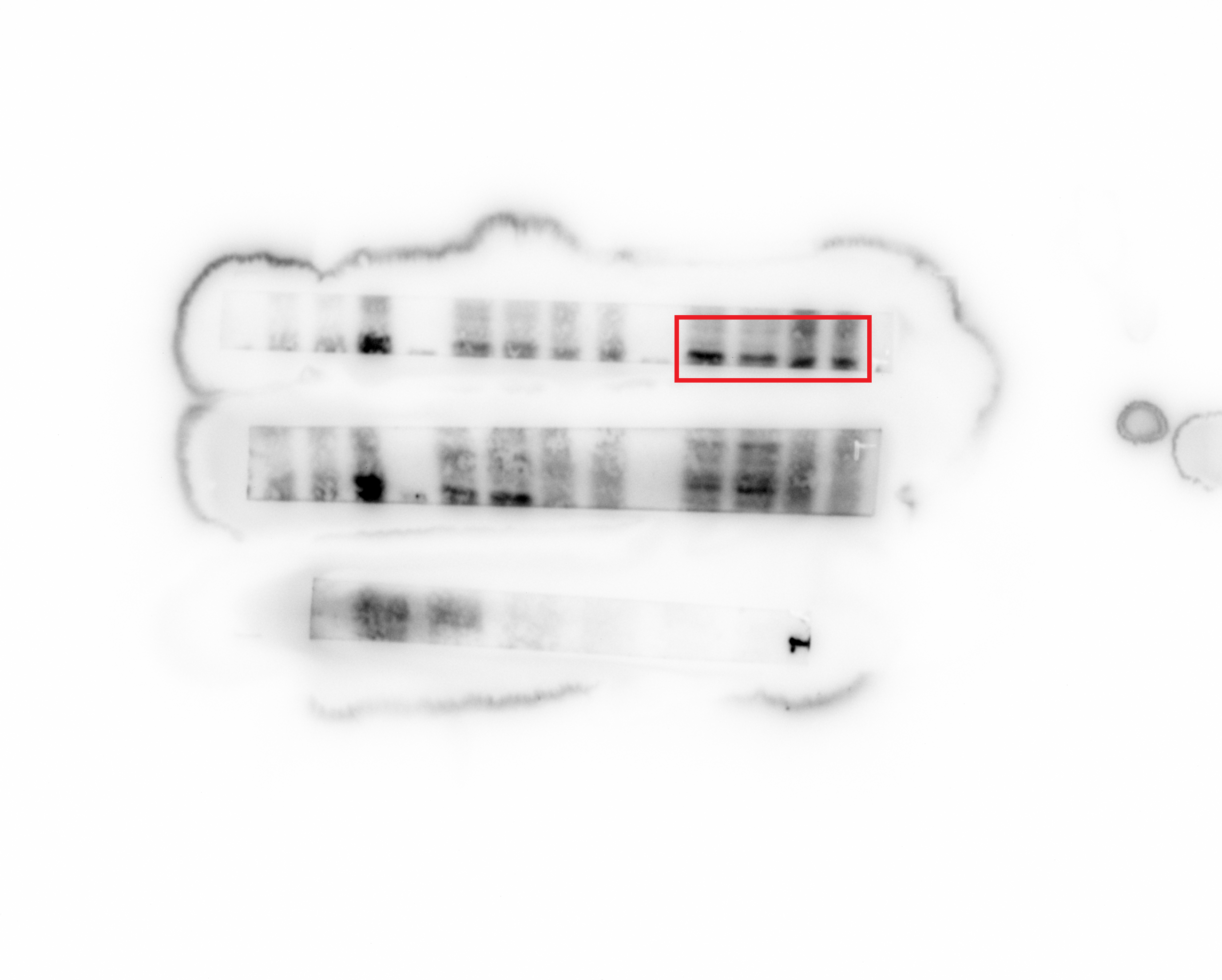

Supplement: Supplementary file 2 [file DataSheet_1.zip › Raw western blot images/Figure 4C/NLRP3.tiff]

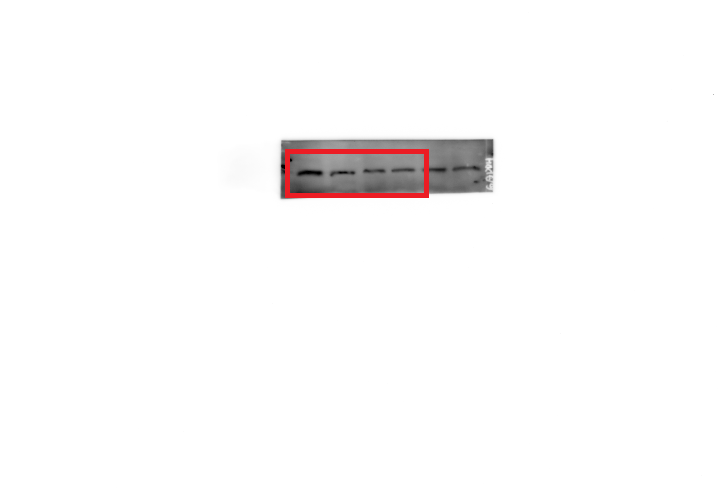

Supplement: Supplementary file 2 [file DataSheet_1.zip › Raw western blot images/Figure 5B/Adcy3.tif]

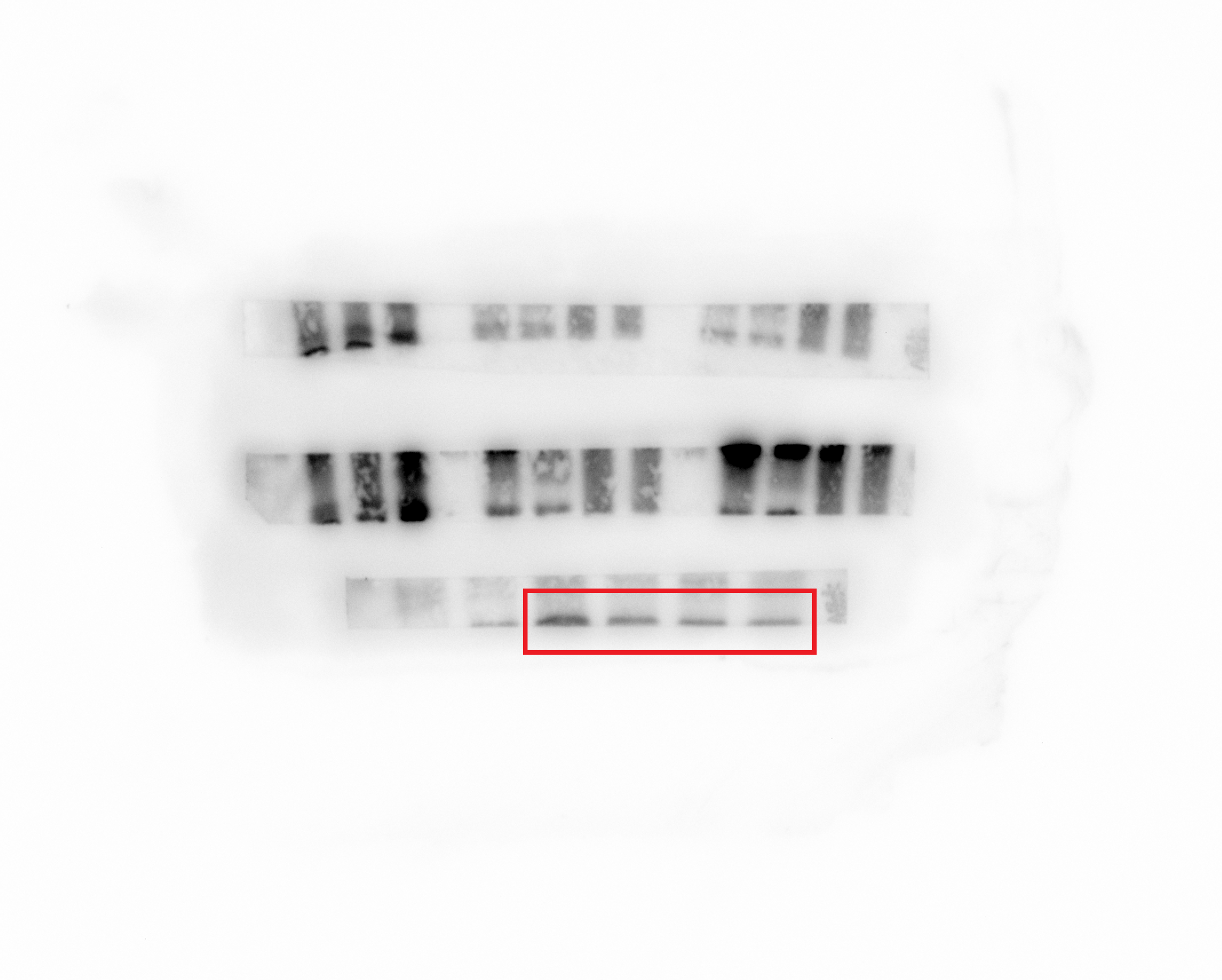

Supplement: Supplementary file 2 [file DataSheet_1.zip › Raw western blot images/Figure 5B/ASC.tiff]

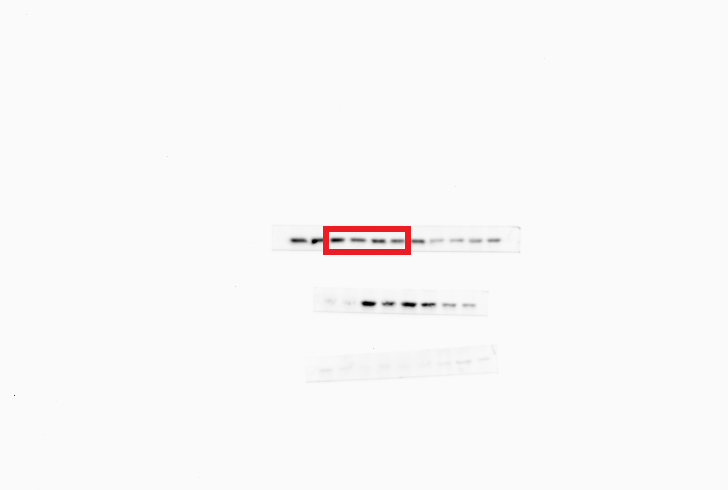

Supplement: Supplementary file 2 [file DataSheet_1.zip › Raw western blot images/Figure 5B/Caspase-1.tiff]

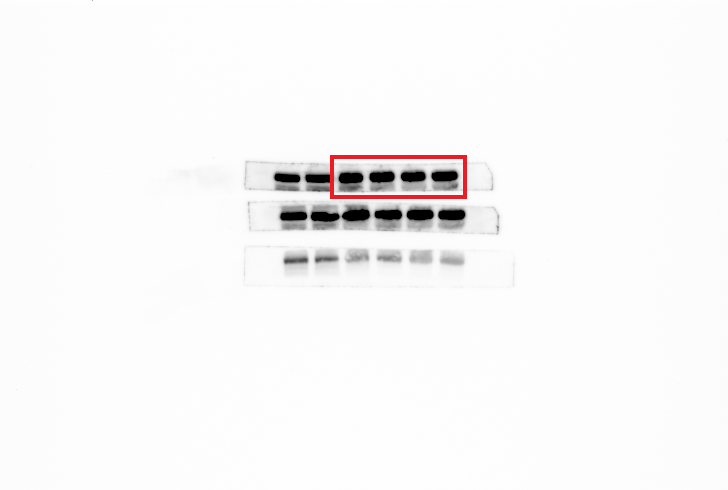

Supplement: Supplementary file 2 [file DataSheet_1.zip › Raw western blot images/Figure 5B/GAPDH.tif]

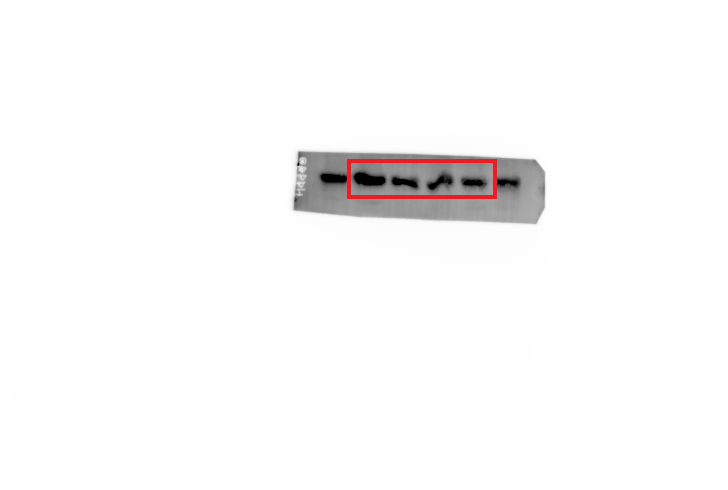

Supplement: Supplementary file 2 [file DataSheet_1.zip › Raw western blot images/Figure 5B/GSDMD.tif]

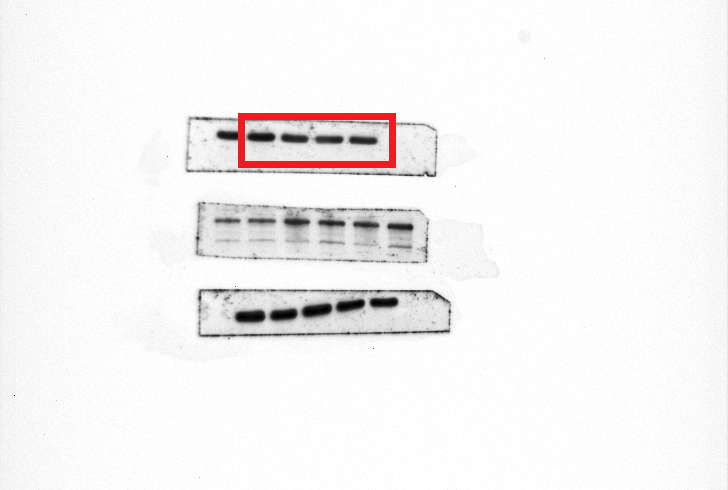

Supplement: Supplementary file 2 [file DataSheet_1.zip › Raw western blot images/Figure 5B/NEK7.tif]

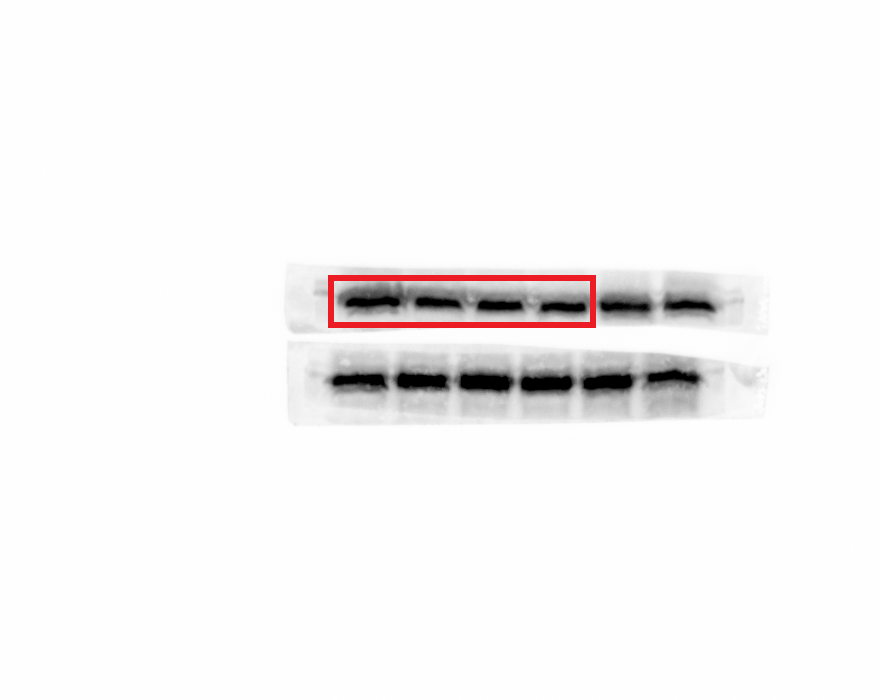

Supplement: Supplementary file 2 [file DataSheet_1.zip › Raw western blot images/Figure 5B/NLRP3.tiff]

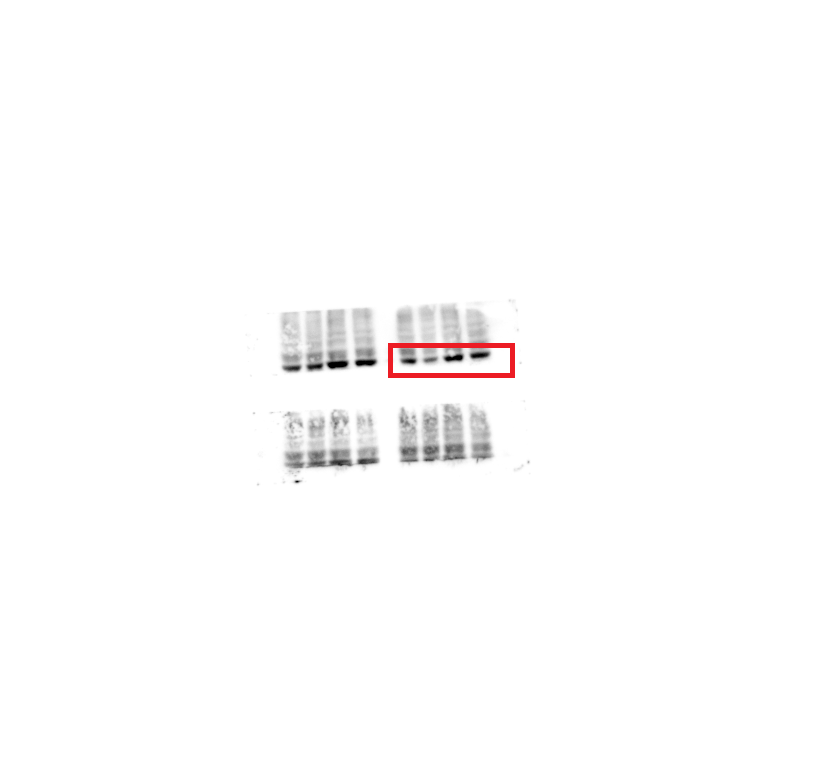

Supplement: Supplementary file 2 [file DataSheet_1.zip › Raw western blot images/Figure 6C/Adcy3.tiff]

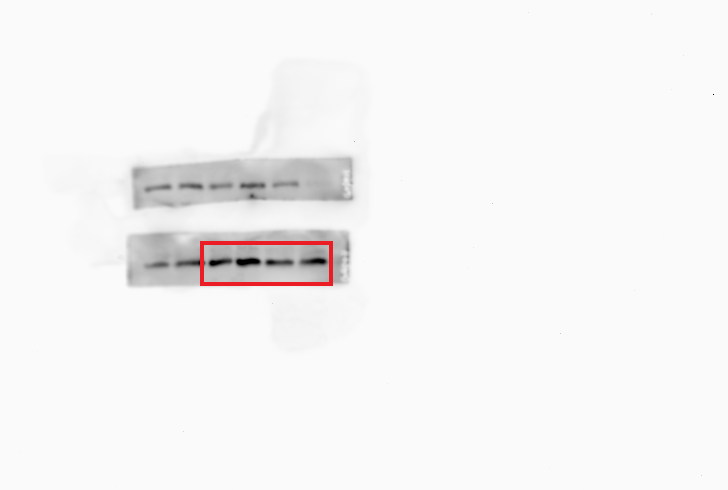

Supplement: Supplementary file 2 [file DataSheet_1.zip › Raw western blot images/Figure 6C/Arg-1.tif]

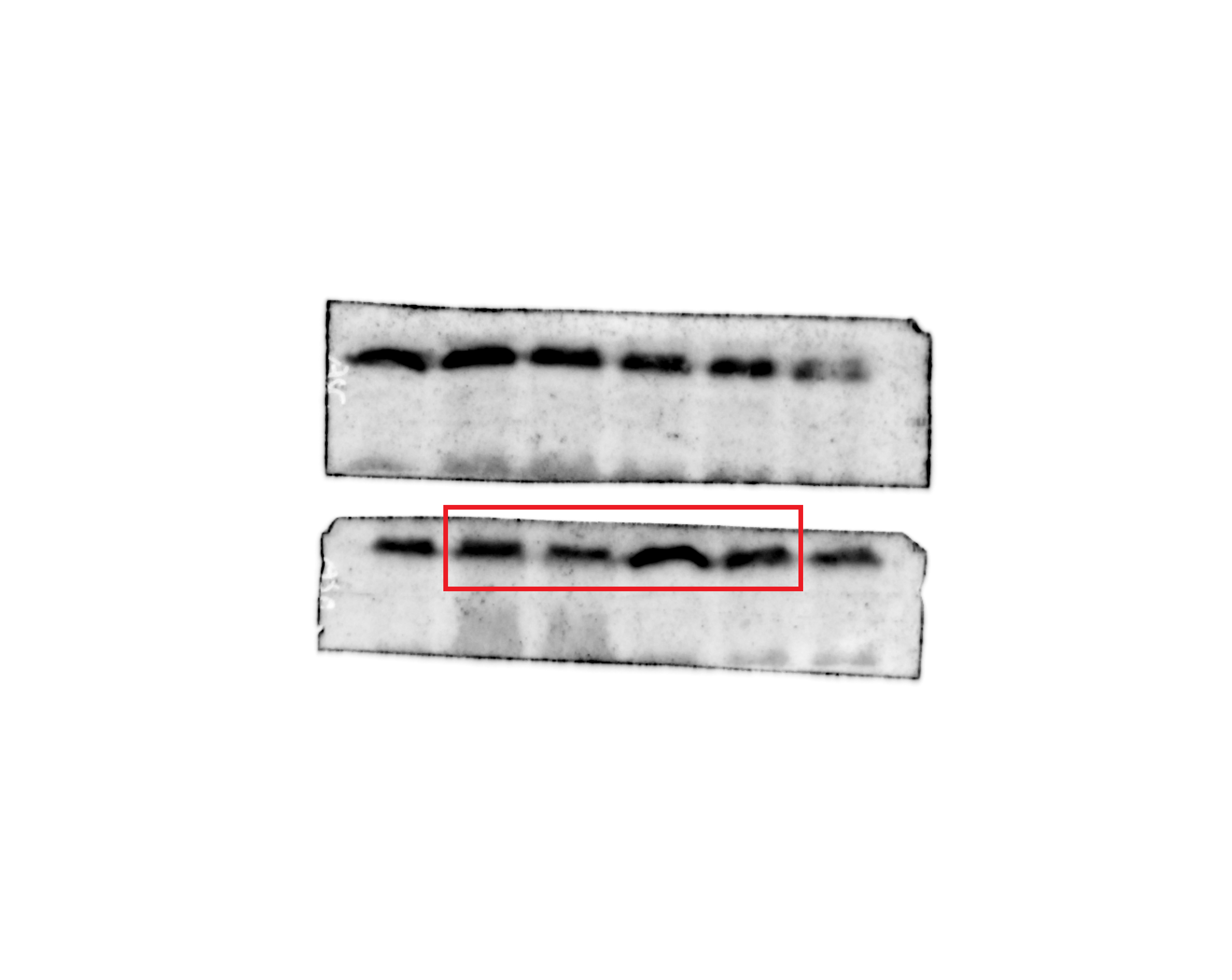

Supplement: Supplementary file 2 [file DataSheet_1.zip › Raw western blot images/Figure 6C/ASC.tiff]

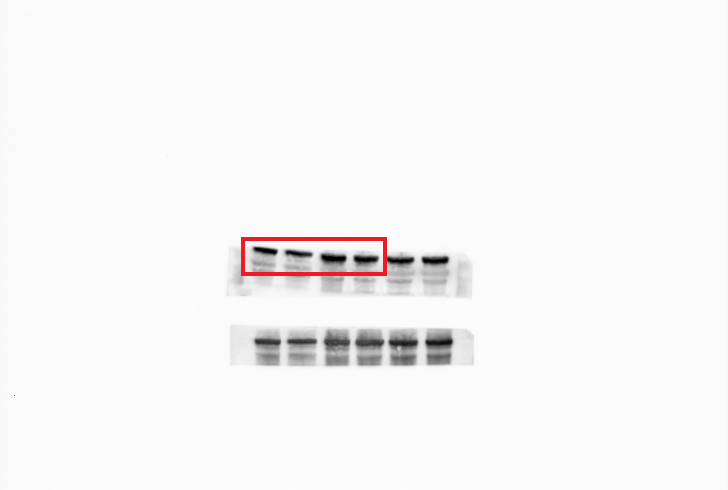

Supplement: Supplementary file 2 [file DataSheet_1.zip › Raw western blot images/Figure 6C/Caspase-1.tif]

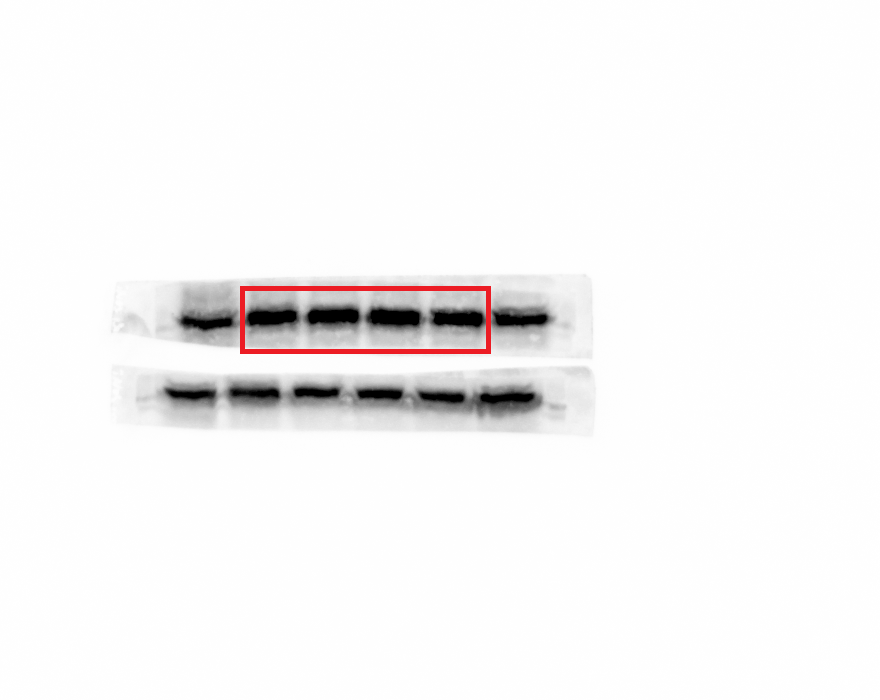

Supplement: Supplementary file 2 [file DataSheet_1.zip › Raw western blot images/Figure 6C/GAPDH.tiff]

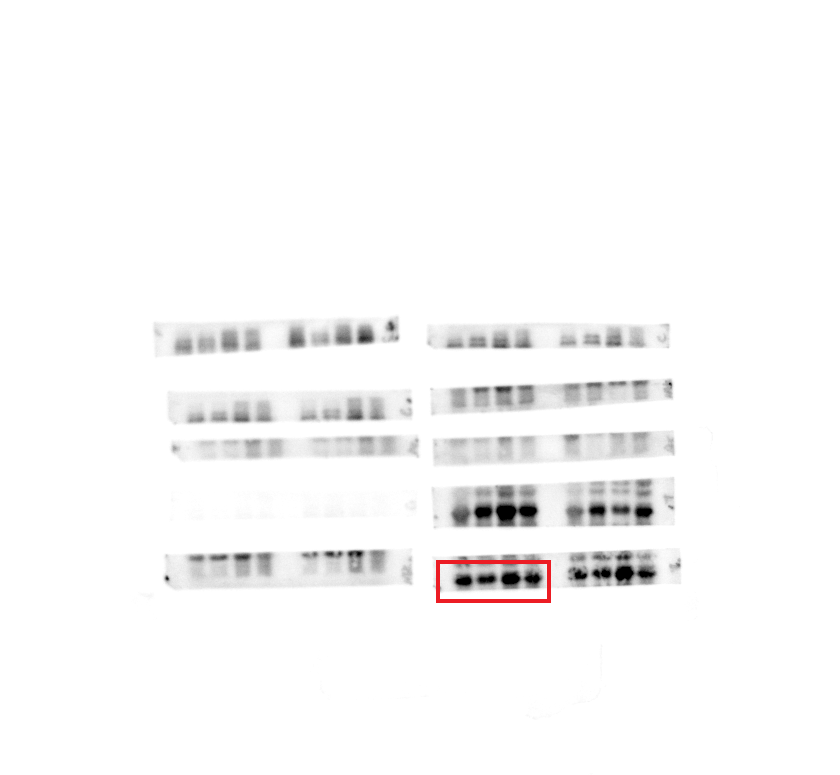

Supplement: Supplementary file 2 [file DataSheet_1.zip › Raw western blot images/Figure 6C/GSDMD.tiff]

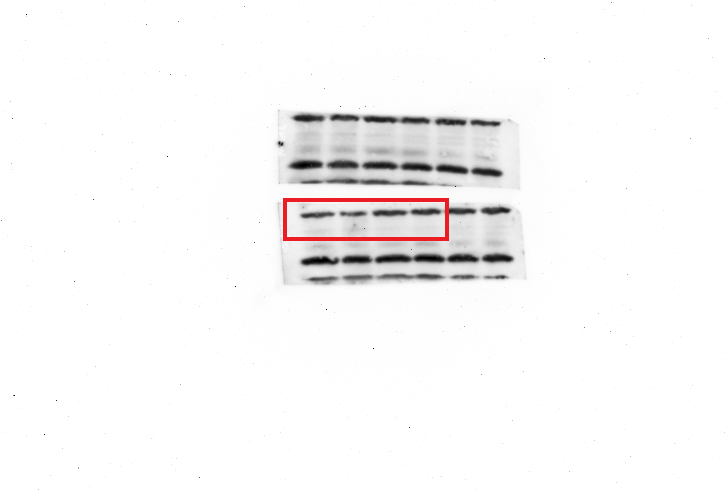

Supplement: Supplementary file 2 [file DataSheet_1.zip › Raw western blot images/Figure 6C/iNOS.tif]

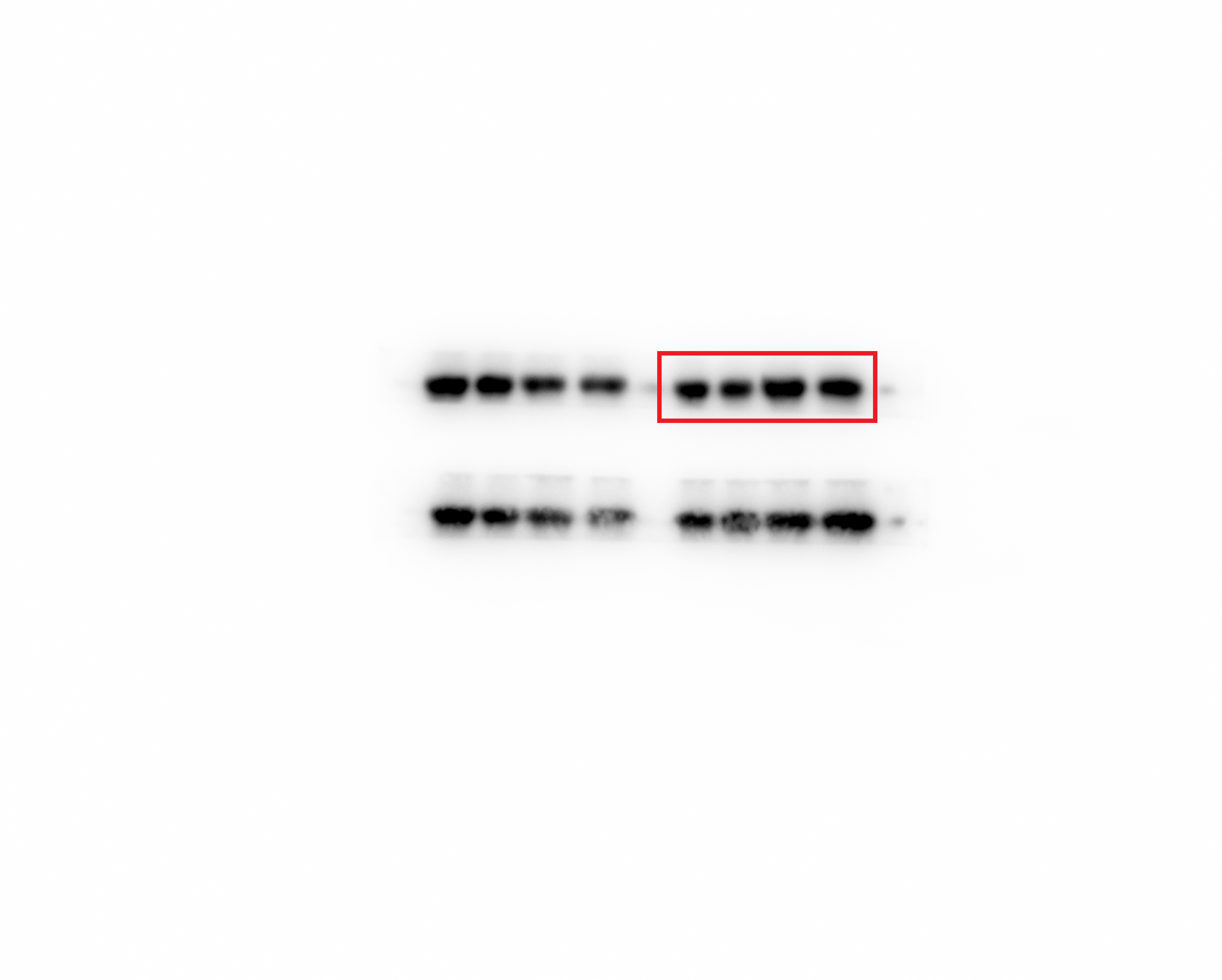

Supplement: Supplementary file 2 [file DataSheet_1.zip › Raw western blot images/Figure 6C/NEK7.tiff]

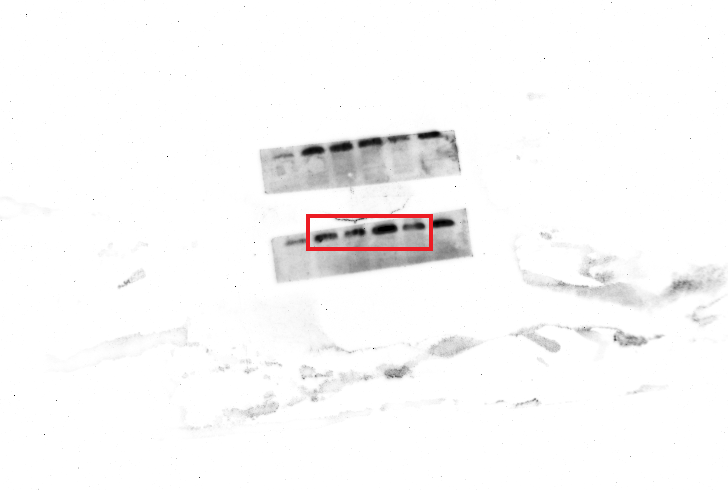

Supplement: Supplementary file 2 [file DataSheet_1.zip › Raw western blot images/Figure 6C/NLRP3.tiff]

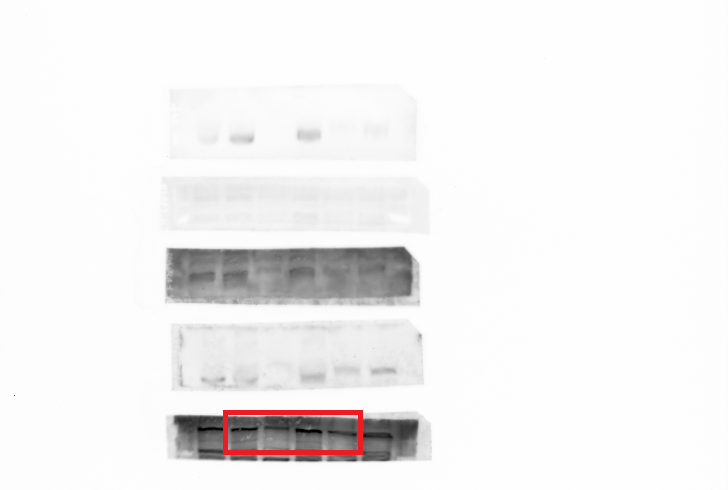

Supplement: Supplementary file 2 [file DataSheet_1.zip › Raw western blot images/Figure 7B/Adcy3.tif]

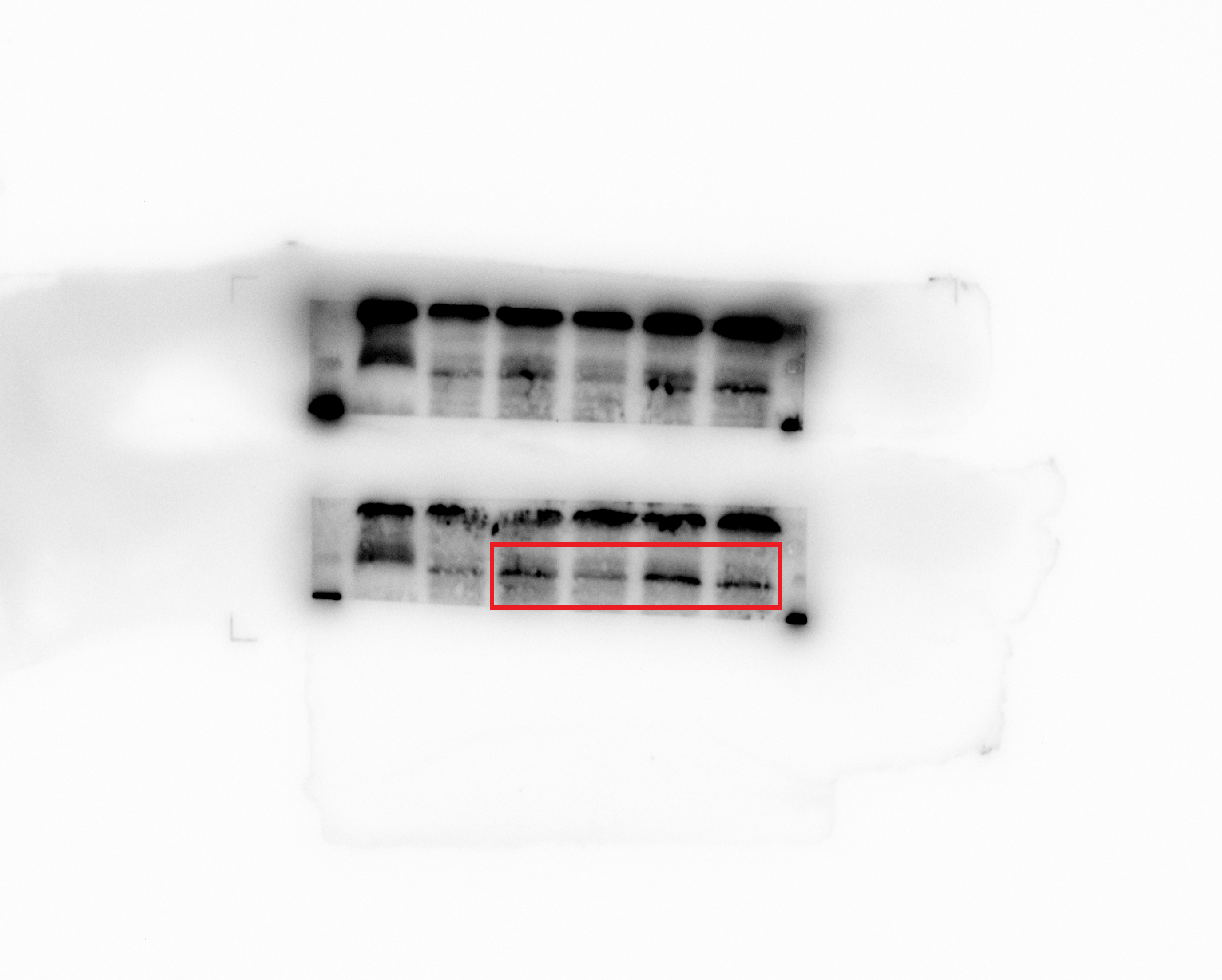

Supplement: Supplementary file 2 [file DataSheet_1.zip › Raw western blot images/Figure 7B/ASC.tiff]

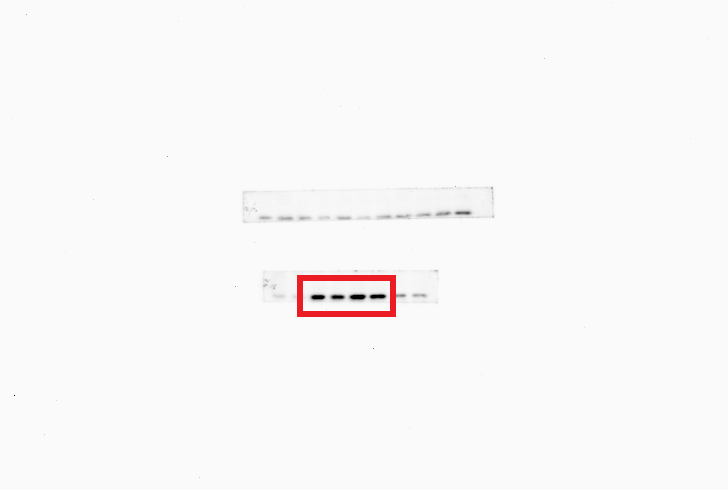

Supplement: Supplementary file 2 [file DataSheet_1.zip › Raw western blot images/Figure 7B/Caspase-1.tiff]

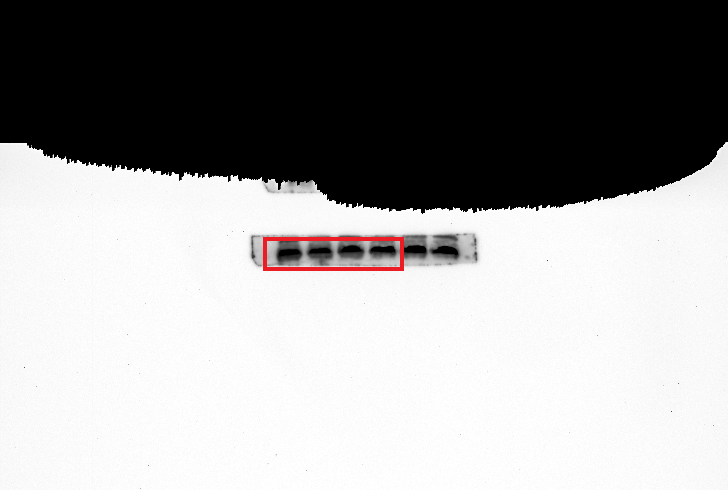

Supplement: Supplementary file 2 [file DataSheet_1.zip › Raw western blot images/Figure 7B/GAPDH.tif]

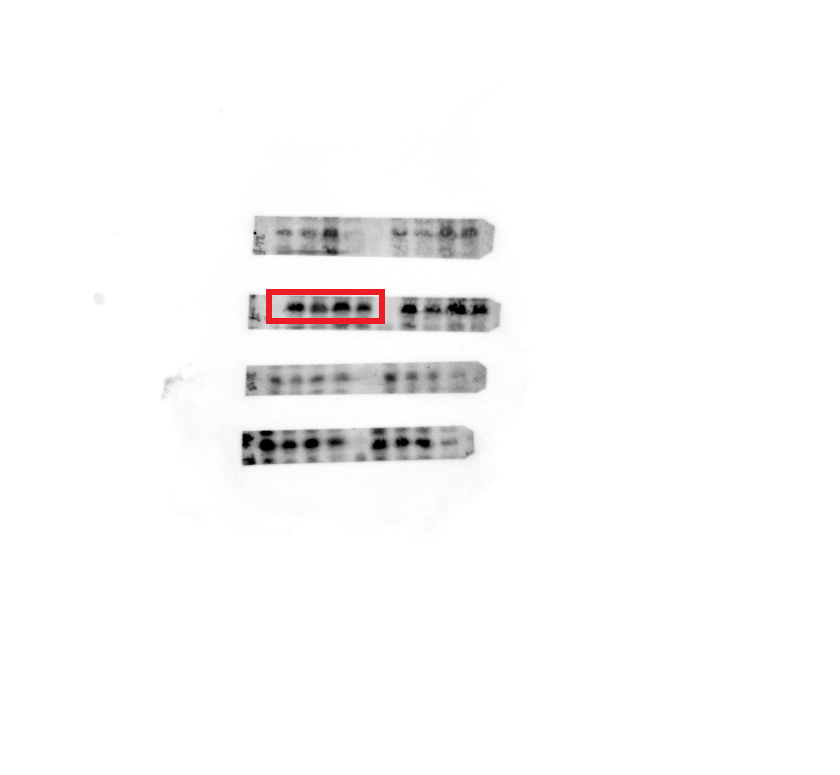

Supplement: Supplementary file 2 [file DataSheet_1.zip › Raw western blot images/Figure 7B/GSDMD.tiff]

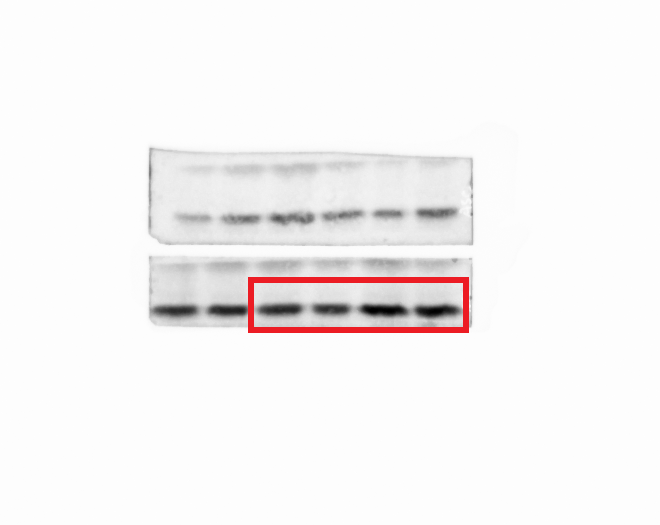

Supplement: Supplementary file 2 [file DataSheet_1.zip › Raw western blot images/Figure 7B/NEK7.tiff]

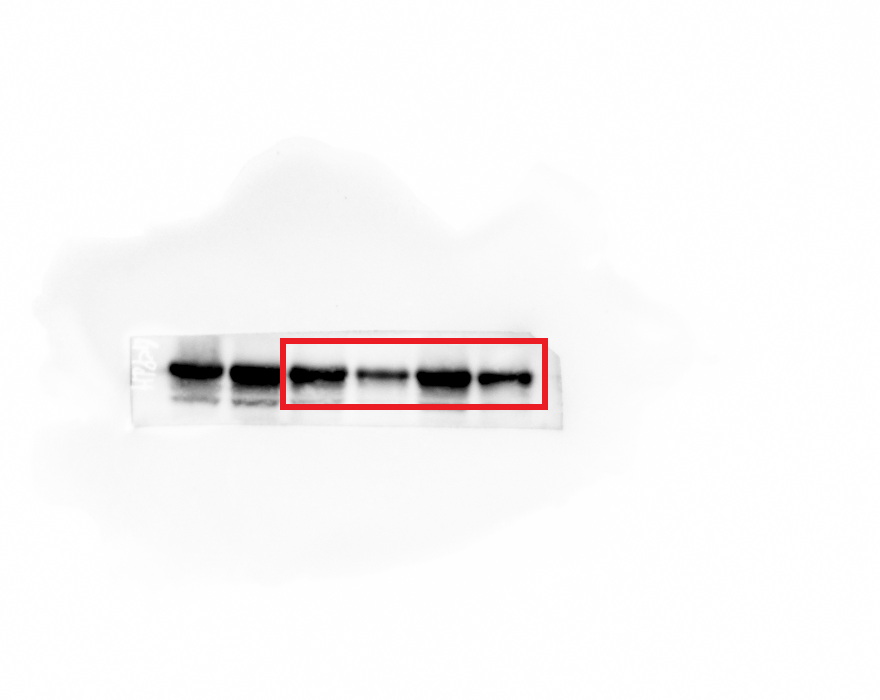

Supplement: Supplementary file 2 [file DataSheet_1.zip › Raw western blot images/Figure 7B/NLRP3.tiff]

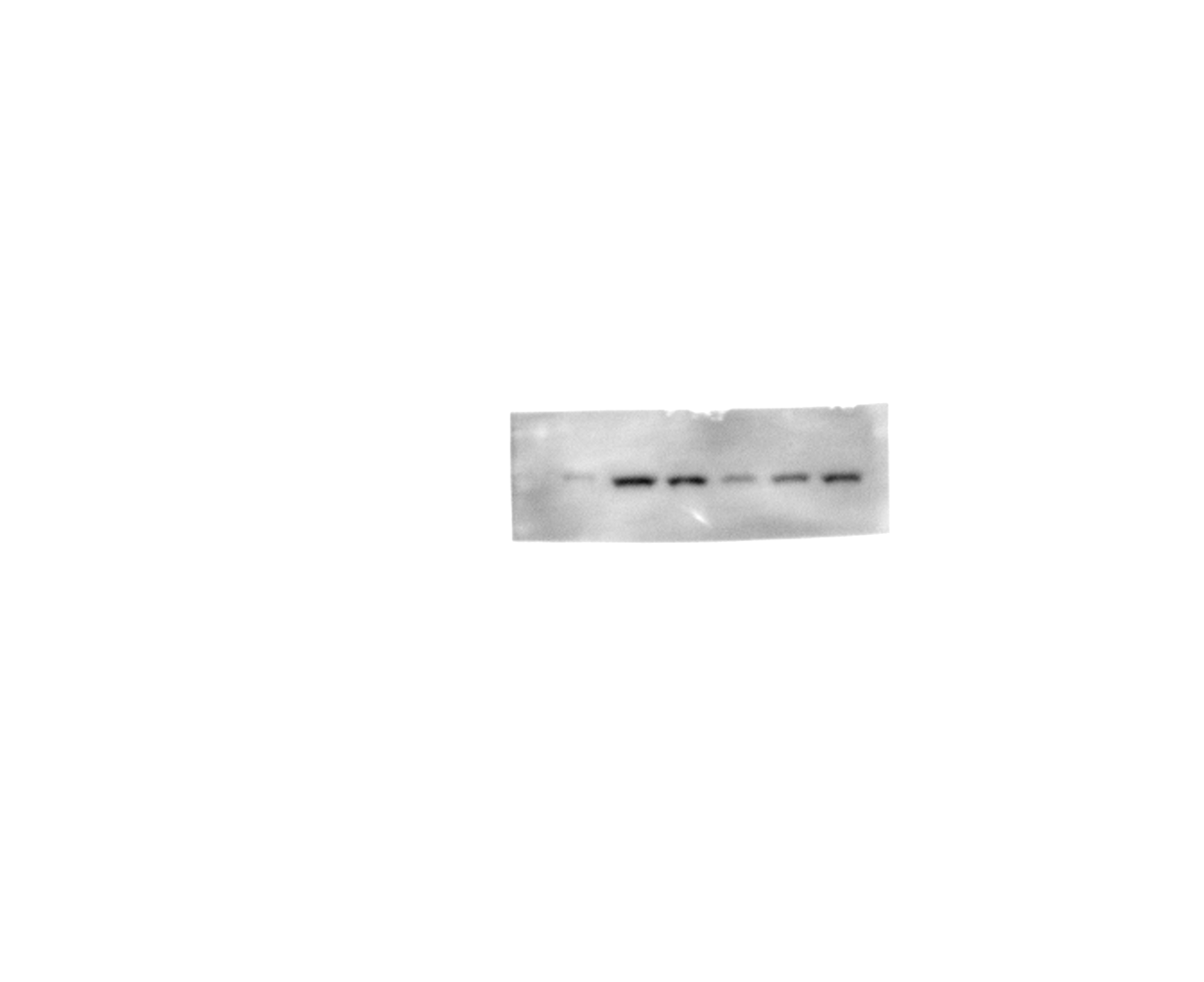

Supplement: Supplementary file 2 [file DataSheet_1.zip › Raw western blot images/Supplemental figure 3B/IL-1a┬.tif]
